# Metabolomic Workflow for the Accurate and High-Throughput Exploration of the Pathways of Tryptophan, Tyrosine, Phenylalanine, and Branched-Chain Amino Acids in Human Biofluids

**DOI:** 10.1021/acs.jproteome.1c00946

**Published:** 2022-04-05

**Authors:** Andrea Anesi, Kirsten Berding, Gerard Clarke, Catherine Stanton, John F. Cryan, Noel Caplice, R. Paul Ross, Andrea Doolan, Urska Vrhovsek, Fulvio Mattivi

**Affiliations:** † Unit of Metabolomics, Department of Food Quality and Nutrition, Research and Innovation Centre, Fondazione Edmund Mach (FEM), 38010 San Michele all’Adige, Italy; ‡ APC Microbiome Ireland, University College Cork, T12 YT20 Cork, Ireland; § Department of Psychiatry and Neurobehavioural Sciences, University College Cork, T12 YT20 Cork, Ireland; ∥ Department of Anatomy and Neuroscience, University College Cork, T12 YT20 Cork, Ireland; ⊥ Centre for Research in Vascular Biology, University College Cork, T12 YT20 Cork, Ireland; # Atlantia Food Clinical Trial, Blackpool, T23 R50R Cork, Ireland; ∇ Department of Cellular, Computational and Integrative Biology (CIBIO), University of Trento, 38123 Trento, Italy

**Keywords:** tryptophan and tyrosine metabolism, gut microbiota metabolites, host-gut microbiota cometabolism, mass spectrometry, plasma, serum, urine

## Abstract

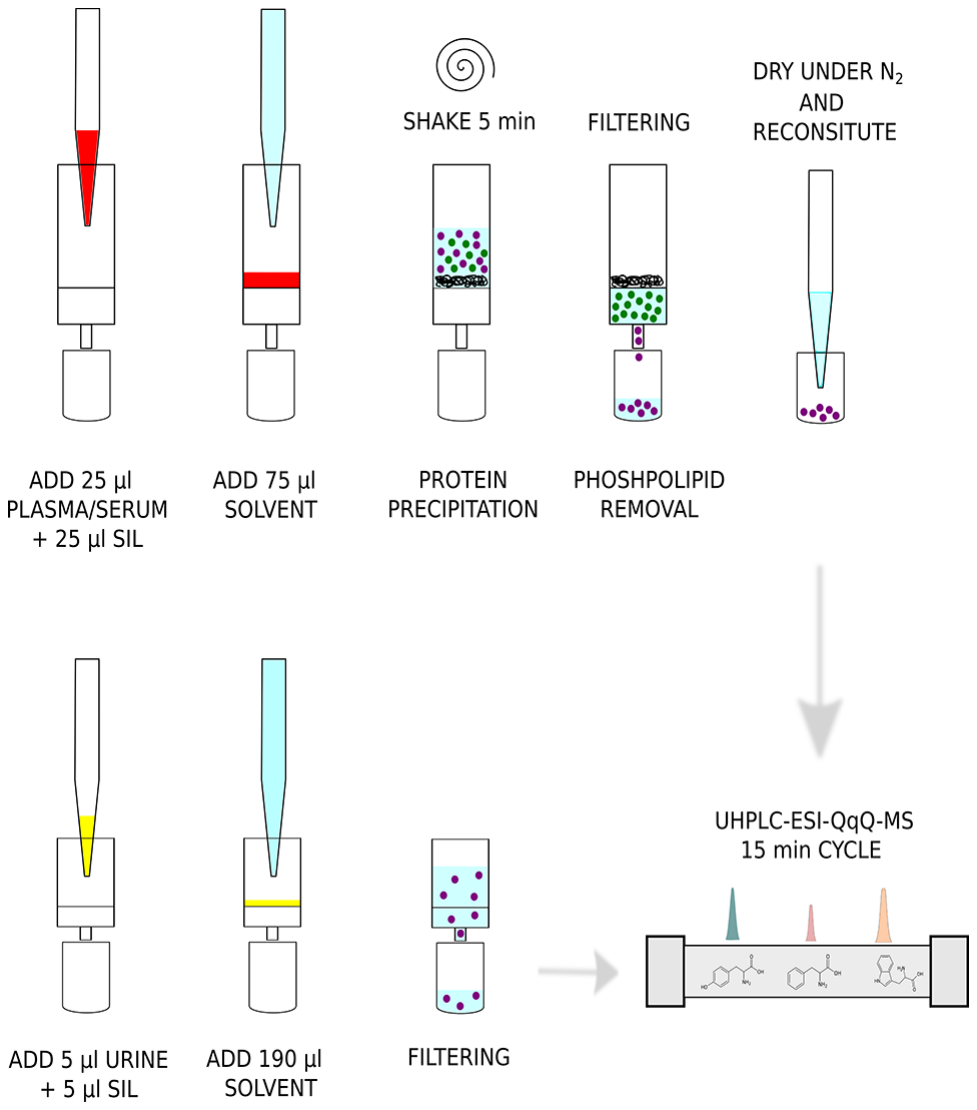

The modulation of host and dietary metabolites by gut microbiota (GM) is important for maintaining correct host physiology and in the onset of various pathologies. An ultrahigh-performance liquid chromatography-electrospray ionization-tandem mass spectrometry method was developed for the targeted quantitation in human plasma, serum, and urine of 89 metabolites resulting from human-GM cometabolism of dietary essential amino acids tryptophan, tyrosine, and phenylalanine as well as branched-chain amino acids. Ninety-six-well plate hybrid-SPE enables fast clean-up of plasma and serum. Urine was diluted and filtered. A 15 min cycle enabled the acquisition of 96 samples per day, with most of the metabolites stable in aqueous solution for up to 72 h. Calibration curves were specifically optimized to cover expected concentrations in biological fluids, and limits of detection were at the order of ppb. Matrix effects were in acceptable ranges, and analytical recoveries were in general greater than 80%. Inter and intraday precision and accuracy were satisfactory. We demonstrated its application in plasma and urine samples obtained from the same individual in the frame of an interventional study, allowing the quantitation of 51 metabolites. The method could be considered the reference for deciphering changes in human-gut microbial cometabolism in health and disease. Data are available via Metabolights with the identifier MTBLS4399.

## Introduction

The human gut is colonized by more than 100 trillion microbes, comprising archaea, bacteria, fungi, protozoa, and viruses.^1,2^ In recent years, scientific research has focused on the relationship between gut microbiota (GM) and host (patho)physiology, and increasing evidence supported the impact of GM-derived metabolites on human health. Diabetes,^3−5^ inflammatory bowel disease (IBD),^6,7^ irritable bowel syndrome (IBS),^8−10^ obesity,^1,11^ cancer,^12,13^ cardiovascular disease,^6,14,15^ and psychiatric disorders^16,17^ are examples of pathologies connected to an altered composition of GM or dysbiosis. GM-derived metabolites can be divided into three categories according to their origin: (1) metabolites biosynthesized de novo by GM, as branched-chain amino acids (BCAAs), biogenic amines (tyramine (TYRA)) and histamine (HSM), and vitamins; (2) metabolites produced by GM by transformation of dietary components, as short-chain fatty acids, tyrosine (TYR), and tryptophan (TRP) catabolites and trimethylamine-*N-*oxide (TMAO); and (3) metabolites produced by the host and modified by GM, as secondary bile acids.^2,18−20^

GM catabolism of dietary TRP has attracted particular interest from the scientific community given its diverse biological activities connected to brain activity and immune response.^2,21−25^ The aryl hydrocarbon receptor, a cytosolic transcription factor involved in xenobiotic metabolism, is an important regulator of host immunity and inflammation, and its activity is modulated by several ligands produced by GM.^26,27^ The concept of “microbiota-gut-brain axis” was introduced to explain the communication between the human brain and GM. The crosstalk is bidirectional: the brain modulates digestion and appetite, while GM can modulate various brain processes, for example through the release of a set of metabolites that impact brain function and development.^28−30^ TRP is an essential amino acid introduced with the diet; TRP absorption occurs in the small intestine, but 5–10% of it reaches the colon, where it can be metabolized by GM.^31^ About 95% of ingested TRP enters the kynurenine (KYN) pathway, resulting in the production of several important intermediates, such as the neuroprotective kynurenic acid (KA) or the neurotoxic quinolinic acid (QA)^32,33^ (Figure S1). Minor routes of TRP catabolism lead to the production of serotonin (5-HT) through the hydroxylation pathway, tryptamine (TRYP) through the decarboxylation pathway, and indole-3-pyruvic acid (IPYR) through the transamination pathways^34,35^ (Figure S2). GM catabolize TRP into indole (IND) via tryptophanase enzyme, an intermediate for the production of GM-derived INDs, including indole-3-carboxaldehyde (ICARB), indole-3-lactic acid (ILA), and indole-3-propionic acid (IPA).^27,36^

While these catabolic pathways have been widely studied, less is known about the influence of GM on phenylalanine (PHE) and TYR metabolism and on metabolites derived by host-GM cometabolism.^19,37−39^ For example, *Clostridium sporogenes*, a commensal bacterium found in the gut, is able to produce IPA starting from TRP. Similar oxidative and reductive pathways are responsible for the production of the PHE and TYR propionic acid derivatives phenylacetic acid (PAA), 4-hydroxyphenylacetic acid (4OH-PAA), phenyl propionic acid (PPA), and 4-hydroxyphenylpropionic acid (4OH-PPA).^19,40^ TYR is degraded into *p*-cresol and other metabolites; in the liver, *p-*cresol is conjugated to form *p-*cresol sulfate (PCS) and *p*-cresol-glucuronide (PCG),^41^ known to be uremic toxins (Figure S3). PHE is metabolized into PAA and benzoic acid (BA) that are conjugated in the liver to form phenylacetylglutamine (PAGLU), phenylacetylglycine (PAGLY), and hippuric acid (HIP), the most abundant urinary metabolites of host-microbial origin. These metabolites can also be derived from catabolism of dietary flavonoids and anthocyanins,^42−46^ as well as from the catabolism of dietary chlorogenic acid, quinic acid, or shikimic acid, via cyclohexanecarboxylic acid^47^ (Figure S4). On the other hand, BCAAs, such as l-methionine (MET), l-valine (VAL), l-isoleucine (ILE) and l-leucine (LEU), HSM, and TMAO, have been correlated with the onset and progression of inflammation, intestinal pathologies, depression, and cancer.^18,48^ Clinical metabolomics is the branch of analytical chemistry that aims at investigating metabolites in health and disease, providing mechanisms of diseases or information on the organism’s physiological status. Untargeted and targeted metabolomics are the two most commonly used approaches.^49,50^ Untargeted approaches (LC–MS) are used to monitor thousands of known and unknown metabolites at the same time, aiming at discovering new biomarkers. However, data are semiquantitative, and there is a lack of any information regarding analytical precision, accuracy, linearity, and limits of detection and quantitation.^49^

These facts limit the transferability of untargeted metabolomics findings to real-life practice. It is important to define normal and pathological metabolite concentration ranges for diagnostic purposes, as well as absolute concentrations are required in case biomarkers are used in clinical practice.^50^ This can be achieved by targeted approaches (LC–MS/MS). These methods cover a limited number of preselected metabolites and require times for their development and validation but provide robust data and absolute metabolite concentrations that, in a personalized medicine perspective, can decipher the efficacy of medical treatments or nutritional interventions, as well as to evaluate chronical disease progression.^50^ For example, a multicenter, prospective observational CANONIC study on the pathogenesis of acute decompensation (AD) and acute-on-chronic liver failure in cirrhosis has recently demonstrated that changes in metabolites arising from the KYN pathway correlated with disease severity, clinical outcome, and mortality.^51^ To date, liquid chromatography–tandem mass spectrometry (LC–MS/MS) methods in the field of host-microbial cometabolism cover a limited number of metabolites, mainly those of TRP catabolism via KYN or hydroxylation pathways, or are limited to one biological fluid or have only been validated in small population cohorts.^52−56^ Considering the importance of the microbiota in human physiology and the move to understand mechanisms of the microbiota-human physiology crosstalk, there is the need to include advanced, high-throughput methods that increase the number of metabolites that can be screened in a single analysis and are capable of analyzing multiple biological fluids in clinical studies. The methodology reported in this paper is a substantial improvement of the work previously published by our group,^56^ now allowing the quantitation of 89 metabolites of the host-microbiota cometabolism, and to study the biological fate of essential and BCAAs. The method is multicompartment and was designed to handle the three most used biological fluids, human plasma, serum, and urine. The method was designed for low sample requirement (25 μL of plasma and serum and 5 μL of urine), fast processing, high throughput (15 min ultrahigh-performance liquid chromatography-electrospray ionization-tandem mass spectrometry (UHPLC-ESI-MS/MS) run time), and high reproducibility. The method was tested on plasma and urine samples obtained from the same individuals in the frame of an intervention trial to evaluate the efficacy of a probiotic on blood lipids and cholesterol levels in healthy mildly hypercholesterolaemic adults. The method was able to determine the metabolite concentration ranges of 51 metabolites and to detect changes between control and placebo groups, demonstrating its applicability to large clinical cohort studies.

## Experimental Section

### Chemicals and Reagents

Chemical standards listed in Table 1 were purchased from Sigma-Aldrich (Milan, Italy), except for 3-hydroxyanthranilic acid, 3-methoxy-*p*-tyramine (3ME-TYRA), 5-hydroxyindole-3-acetic acid (5OH-IAA), 5-hydroxy-l-tryptophan (5OH-TRP), 6-sulfathoxymelatonin (6-SME), dopamine HCl (DA), homovanillic acid (HVA), indole-3-acetamide (IACT), indole-3-acetonitrile (IACN), KA, *N*-acetyl-5-hydroxytryptamine (NAC-5HT), serotonin HCl (5-HT), and xanthurenic acid (XA), which were purchased from Spectra 2000 (Rome, Italy), 5-hydroxytryptophol (5OH-IET), indoyl-3-acryloylglycine (IAG), and 4-hydroxyphenylpropionylglycine (4OH-PPG) which were purchased from ChemSpace (Riga, Latvia), and PAGLU and PCS, which were purchased from LGC (Milan, Italy). Stable isotope-labeled internal standards (SILs) were purchased from Spectra 2000 (Rome, Italy), except for hippuric acid-D_5_ (HIP-D_5_) and trans-cinnamic acid-D_5_ (CA-D_5_) which were purchased from CDN isotopes (Rome, Italy), and l-isoleucine-D_10_ (ILE-D_10_), l-leucine-D_10_ (LEU-D_10_), and xanthurenic acid-D_4_ (XA-D_4_), which were purchased from LGC (Milan, Italy). Human plasma and human serum were obtained from Sigma-Aldrich (Milan, Italy). HMDB^57^ or PubChem^58^ accession numbers are reported in Table S1.

**Table 1:** Chromatographic Retention Time and MS/MS Parameters for Tested Metabolites^a^

name (abbreviation)	RT (min)	parent *m*/*z*	quantifier *m*/*z* (Q)	qualifier *m/z* (q)	SIL	ESI	DP(V)	EP(V)	CE Q/q(V)	CXP(V)
histamine (HSM)	0.72	112.1	68.1	95.0	MET-D_4_	+	30	10	29/19	15
histidine (HSD)	0.8	156.2	110.0	93.0	MET-D_4_	+	30	10	19/30	10
trimethylamine (TMA)	0.84	60.0	44.0	–	MET-D_4_	+	30	12	25	12
γ-aminobutyric acid (GABA)	0.86	104.1	87.1	69.0	MET-D_4_	+	15	15	14/20	10
trimethylamine-*N*-oxide (TMAO)	0.88	76.2	58.2	42.1	MET-D_4_	+	70	15	58/60	15
norepinephrine (NOR)	0.95	170.3	107.0	135.0	MET-D_4_	+	20	15	26/20	15
epinephrine (EPI)	1.11	184.3	166.2	107.0	MET-D_4_	+	30	15	14/30	15
l-valine (VAL)	1.20	118.1	72.0	55.0	MET-D_4_	+	25	10	15/28	15
picolinic acid (PA)	1.23	124.0	78.0	106.0	MET-D_4_	+	15	15	26/15	15
nicotinic acid (NA)	1.38	124.0	80.1	78.0	MET-D_4_	+	25	8	30/23	15
l-methionine-D_4_ (MET-D_4_)	1.48	154.2	108.1	63.1		+	25	15	14/28	15
l-methionine (MET)	1.50	150.3	133.2	104.0	MET-D_4_	+	20	15	12/14	15
quinolinic acid (QA)	1.61	168.2	78.0	124.2	MET-D_4_	+	20	15	30/15	15
l-Dopa (l-DOPA)	1.62	198.3	152.1	181.1	MET-D_4_	+	35	15	18/14	15
2-aminophenol (2 AM)	1.64	110.0	65.0	92.1	MET-D_4_	+	35	15	29/21	10
dopamine- D_4_ (DA-D_4_)	1.65	158.2	95.0	141.1		+	25	15	33/14	15
dopamine (DA)	1.67	154.3	91.1	119.1	DA-D_4_	+	20	15	31/25	15
3-hydroxykynurenine (3OH-KYN)	1.93	225.3	208.2	162.1	KYN-D_4_	+	20	15	13/25	15
l-isoleucine-D_10_ (ILE-D_10_)	2.00	142.3	96.1	78.4		+	15	15	16/27	10
l-isoleucine (ILE)	2.02	132.1	86.2	69.2	ILE-D_10_	+	30	15	14/25	10
l-tyrosine-D_4_ (TYR-D_4_)	2.02	186.2	140.1	169.2		+	30	15	20/14	15
l-tyrosine (TYR)	2.05	182.1	91.0	119.0	TYR-D_4_	+	25	15	35/31	15
tyramine (TYRA)	2.18	138.2	121.0	77.0	TYR-D_4_	+	25	15	14/36	15
l-leuicne-D_10_ (LEU-D_10_)	2.20	142.3	96.1	48.0		+	15	15	36/14	15
l-leucine (LEU)	2.21	132.1	86.2	44.2	LEU-D_10_	+	30	15	14/33	15
serotonin-D_4_ (5-HT-D_4_)	2.57	181.3	118.0	134.2		+	25	15	40/45	15
serotonin (5-HT)	2.60	177.2	160.1	115.2	5-HT-D_4_	+	25	10	16/35	15
3-methoxy-*p*-tyramine (3ME-TYRA)	2.61	168.3	151.1	91.0	TYR-D_4_	+	20	15	14/32	15
5-hydroxy-l-tryptophan (5OH-TRP)	2.65	221.3	162.2	134.2	TRP-D_5_	+	25	15	25/34	15
*N*-methylserotonin (ME-5HT)	2.72	191.3	160.2	148.1	5-HT-D_4_	+	20	10	1818	15
l-kynurenine-D_4_ (KYN-D_4_)	2.80	213.2	196.3	150.0		+	25	15	13/25	15
l-kynurenine (KYN)	2.82	209.3	146.0	94.0	KYN-D_4_	+	20	10	25/19	15
l-phenylalanine (PHE)	2.87	166.2	103.0	77.0	TYR-D_4_	+	35	10	36/49	15
4-hydroxyphenylacetylglycine (4OH-PAG)	3.45	210.2	107.0	76.0	TRP-D_5_	+	15	10	28/14	12
3-hydroxyanthranilic acid (3OH-AA)	3.54	154.2	136.1	80.0	TRP-D_5_	+	30	15	15/34	15
l-tryptophan-D_5_ (TRP-D_5_)	3.69	210.3	192.1	150.0		+	25	15	14/25	15
xanthurenic acid-D_4_ (XA-D_4_)	3.71	210.3	164.2	136.1		+	25	10	27/41	15
l-tryptophan (TRP)	3.72	205.2	188.1	146.0	TRP-D_5_	+	25	10	13/24	15
xanthurenic acid (XA)	3.75	206.0	160.0	132.1	XA-D_4_	+	35	15	25/40	15
3,4-dihydroxyphenylacetic acid-D_5_ (DOPAC-D_5_)	3.77	172.1	128.1	100.0		–	-10	–15	–11/–27	–10
3,4-dihydroxyphenylacetic acid (DOPAC)	3.80	167.1	123.0	95.0	DOPAC-D_5_	–	–10	–15	–12/–26	–10
3-(4-hydroxyphenyl)-lactic acid (4OH-PLA)	3.83	181.2	163.1	135.0	DOPAC-D_5_	–	–35	–10	–16/–21	–10
homovanillic acid sulfate (HVAS)	3.88	261.1	181.0	137.0	DOPAC-D_5_	–	–30	–15	–21/–30	–15
*N*-acetyl-l-tyrosine (NAC-TYR)	3.92	222.1	180.1	163.0	DOPAC-D_5_	–	–25	–15	–18/–28	–15
l-tryptophanol (TROL)	3.93	191.4	130.2	174.0	TRP-D_5_	+	30	15	20/12	15
kynurenic acid-D_5_ (KA-D_5_)	3.98	195.3	149.0	121.1		+	35	15	26/44	15
kynurenic acid (KA)	4.00	190.2	144.1	89.0	KA-D_5_	+	10	10	25/52	15
5-methoxy-l-tryptophan (5ME-TRP)	4.02	235.3	218.2	176.0	TRP-D_5_	+	20	15	15/24	15
tryptamine (TRYT)	4.05	161.0	144.0	117.2	TRP-D_5_	+	30	15	18/32	15
4-hydroxyphenylpropionylglycine (4OH-PPG)	4.10	224.3	107.0	149.2	TRP-D_5_	+	20	12	30/14	15
5-hydroxytryptophol (5OH-IET)	4.10	178.2	160.1	115.0	TRP-D_5_	+	30	15	20/36	15
5-methoxytryptamine (5ME-TRYT)	4.15	191.3	174.3	143.0	TRP-D_5_	+	35	10	15/32	10
6-sulfathoxymelatonin (6-SMEL)	4.20	327.2	161.0	176.1	IS-D_4_	–	–35	–15	–45/–33	–15
5-hydroxyindole-3-acetic acid-D_5_ (5OH-IAA-D_5_)	4.25	197.2	150.1	95.0		+	35	15	25/50	15
5-hydroxyindole-3-acetic acid (5OH-IAA)	4.27	192.2	146.2	91.0	5OH-IAA-D_5_	+	35	10	19/48	15
indoxyl-*β*-glucoside (PLI)	4.28	294.2	160.9	131.0	IS-D_4_	–	–40	–15	–11/–30	–15
*N*-acetyl-5-hydroxytryptamine (NAC-5HT)	4.36	219.2	160.1	132.0	TRP-D_5_	+	25	10	19/35	15
indoxyl-*β*-glucuronide (IBG)	4.37	308.1	112.9	132.1	IS-D_4_	–	–25	–12	–20/–30	–15
indoxyl sulfate-D_4_ (IS-D_4_)	4.42	216.0	80.0	136.1		–	–30	–5	–25/–25	–10
indoxyl sulfate (IS)	4.45	212.0	80.0	132.0	IS-D_4_	–	–30	–15	–30/–26	–15
phenylacetyl-l-glutamine (PAGLU)	4.46	265.3	130.1	91.0	HIP-D_5_	+	20	10	18/44	11
hippuric acid-D_5_ (HIP-D_5_)	4.50	185.2	110.1	82.1		+	10	10	20/43	10
hippuric acid (HIP) PLASMA	4.55	180.2	105.0	77.1	HIP-D_5_	+	10	10	19/42	10
URINE			77.1	51.0					42/77	
l-tryptophan, methyl ester (TRP ME)	4.56	219.2	202.1	160.1	TRP-D_5_	+	25	15	14/25	15
homovanillic acid (HVA)	4.61	181.1	122.0	137.0	HIP-D_5_	–	–35	–10	–19/–11	–10
phenylacetylglycine (PAGLY)	4.78	194.3	91.0	76.0	HIP-D_5_	+	10	12	25/12	10
indole-3-acetyl aspartic acid (IASP)	4.87	291.3	130.1	134.0	HIP-D_5_	+	25	15	30/15	15
3-(4-hydroxyphenyl)-propionic acid (4OH-PPA)	4.88	165.1	121.0	78.9	DOPAC-D_5_	–	–35	–10	–15/–33	–10
*p*-cresol glucuronide (PCG)	4.90	283.1	112.9	175.0	IS-D_4_	–	–30	–8	–18/–14	–13
indole-3-acetamide (IACT)	4.91	175.2	130.2	77.0	HIP-D_5_	+	35	15	22/55	15
l-tryptophan, ethyl ester (TRP EE)	4.95	233.3	216.3	174.2	HIP-D_5_	+	25	15	14/22	15
4-hydroxycinnamic acid (HCA)	5.01	163.0	119.0	93.0	IS-D_4_	–	–10	–11	–22/–40	–10
*p*-cresol sulfate (PCS)	5.03	187.1	80.0	107.0	IS-D_4_	–	–32	–10	–23/–20	–10
indole-3-acetylglutamic acid (IGLUT)	5.03	305.4	130.2	148.1	HIP-D_5_	+	25	10	30/20	15
anthranilic acid (AA)	5.1	138.2	120.0	92.0	HIP-D_5_	+	15	15	15/28	15
*N*-acetyl-l-phenylalanine (NAC-PHE)	5.20	206.1	164.0	147.0	IS-D_4_	–	–25	–15	–17/–22	–15
phenyllactic acid (PLA)	5.20	164.9	147.0	119.0	IS-D_4_	–	–20	–10	–15/–19	–10
*N*-acetyl-l-tryptophan (NAC-TRP)	5.35	245.1	203.1	73.9	IAA-D_5_	–	–30	–15	–18/–23	–15
indole-3-lactic acid (ILA)	5.39	206.3	118.1	130.1	IAA-D_5_	+	35	10	29/40	15
*N*-acetyl-l-tyrosine, ethyl ester (NAC-TYR EE)	5.42	252.2	136.2	178.2	IAA-D_5_	+	15	15	27/16	15
phenylpropionylglycine (PPG)	5.48	208.3	105.0	91.2	IAA-D_5_	+	18	12	24/49	11
indole-3-acryloylglycine (IAG)	5.56	245.2	115.2	142.2	IAA-D_5_	+	30	15	53/38	15
indole-3-carboxylic acid (ICA)	5.59	162.2	116.2	91.0	IAA-D_5_	+	30	15	28/33	10
cinnamoylglycine (CYG)	5.68	206.1	131.0	103.0	IAA-D_5_	+	25	10	18/38	15
indole-3-carboxaldehyde (ICARB)	5.76	146.2	118.1	65.1	IAA-D_5_	+	30	15	34/50	15
melatonin (MEL)	5.78	233.1	174.1	159.0	IAA-D_5_	+	30	15	21/37	15
5-methoxytryptophol (5ME-IET)	5.80	192.3	174.3	130.1	IAA-D_5_	+	25	15	20/49	15
5-methoxyindole-3-acetic acid (5ME-IAA)	5.81	206.3	160.1	117.1	IAA-D_5_	+	30	15	21/47	15
benzoic acid (BA)	5.84	121.0	77.0	92.0	IS-D_4_	–	–30	–10	–15/–35	–10
indole-3-acetic acid-D_5_ (IAA-D_5_)	5.94	181.2	134.1	106.0	IAA-D_5_	+	25	15	23/43	15
indole-3-acetic acid (IAA)	5.96	176.2	130.0	102.9	IAA-D_5_	+	25	15	40/19	15
indole-3-ethanol (IET)	6.00	162.3	144.1	117.1	IAA-D_5_	+	25	15	19/30	15
cinnabarinic acid (CNBA)	6.02	301.1	283.1	265.0	IAA-D_5_	+	30	15	23/42	15
indole-3-acrylic acid (IACR)	6.23	188.2	115.0	170.1	IAA-D_5_	+	30	15	40/18	15
indole-3-propionic acid (IPA)	6.46	190.2	130.1	103.1	IAA-D_5_	+	25	15	24/50	15
*trans*-cinnamic acid-D_5_ (CA-D_5_)	6.58	154.2	107.0	135.0		+	35	15	28/15	15
*trans*-cinnamic acid (CA)	6.60	149.1	77.0	131.1	CA-D_5_	+	25	10	42/13	10
*N*-acetyl-l-tryptophan, ethyl ester (NAC-TRP EE)	6.65	275.3	201.1	229.2	IAA-D_5_	+	25	15	18/13	15
indole-3-butyric acid (IBA)	6.81	204.3	186.1	130.0	IAA-D_5_	+	30	15	19/35	15
indole-3-acetonitrile (IACN)	6.84	157.3	130.1	117.1	IAA-D_5_	+	25	15	15/28	15
indole-3-acetic acid, methyl ester (IAA ME)	7.19	190.2	130.2	103.0	IAA-D_5_	+	25	15	18/48	15
indole (IND)	7.22	118.0	91.0	65.0	IAA-D_5_	+	35	15	30/42	10
indole-3-acetic acid, ethyl ester (IAA EE)	7.50	204.3	130.2	103.0	IAA-D_5_	+	35	15	25/50	15
tryptanthrin (TRPT)	7.55	249.2	130.0	221.0	IAA-D_5_	+	35	15	41/34	17
3-methylindole (SKA)	7.83	132.1	89.0	117.1	IAA-D_5_	+	40	7	53/30	15

a Entries are ordered by increasing retention time (RT). DP: declustering potential; EP: entrance potential; CE: collision energy; CXP: collision-cell exit potential.

LC–MS-grade 2-propanol, acetonitrile (ACN), formic acid (FA), methanol (MeOH), and ammonium formate were purchased from Sigma-Aldrich (Milan, Italy); ultrapure Milli-Q deionized water was obtained from Elix (Merck-Millipore, Milan, Italy). OSTRO 96-well plates (25 mg) were purchased from Waters (Milan, Italy).

### Analytical Protocol

#### Standard Stock Solution Preparation

Stock solutions (1 mg/mL) were prepared by dissolving each standard compound in methanol, except for 3-hydroxykynurenine (3-OHKYN), dopamine-D_4_ HCl (DA-D_4_), epinephrine (EPI), l-kynurenine (KYN), kynurenine-D_4_ (KYN-D_4_), l-dopa, l-isoleucine (ILE), ILE-D_10_, l-leucine (LEU), l-LEU-D_10_, l-methionine-D_4_ (MET-D_4_), norepinephrine (NOREPI), l-tyrosine (TYR), l-tyrosine-D_4_ (TYR-D_4_), and histidine (HIST), which were first dissolved in 1 M HCl and then in MeOH (final ratio 1 M HCl:MeOH 1:9; v:v) and cinnabarinic acid (CNBA), KA, kynurenic acid-D_5_ (KA-D_5_), and l-tryptophanol, which were dissolved DMSO:MeOH 1:1, v/v). XA and XA-D_4_ were first dissolved in DMSO and then diluted to 0.1 mg/mL in MeOH to avoid precipitation. All stock solutions were stored at −20 °C.

#### Dilution of Calibration Standards

Stock solutions for plasma/serum and for urine calibration were prepared by diluting standard stock solution to appropriate concentrations presented in Table S1. Working calibration curves were obtained by dilution in mobile phase A water 0.1% FA.

#### Preparation of Stable Isotope-Labeled Solutions

SILs were diluted in MeOH at specific concentrations, reflecting those of native compounds in plasma/serum or urine. Details are reported in Table S2.

### Extraction of Plasma and Serum Samples by Hybrid-SPE

Human plasma and serum were thawed on ice and vortexed for 15 s before use. Sample aliquots (25 μL) were loaded on OSTRO 96-well plates for protein precipitation and phospholipid removal, and 25 μL of SIL in MeOH were added into each well. According to the manufacturer’s procedures, three volumes of ice-cold ACN 1% FA (75 μL) were added into each well, and plates were shaken on an Eppendorf shaker (Eppendorf, Milan, Italy) for 5 min at 500 rpm. Plates were filtered using a positive-pressure 96-manifold (Water, Milan, Italy) for 5 min at 3 psi. The extraction procedure was repeated one time by adding 75 μL of ice-cold ACN 1% FA. Samples were brought to dryness under a gentle stream of nitrogen (1.5 psi) at 37 °C on a Techne Dr-block DB 3D heater and redissolved two times in 100 μL of water:ACN 95:5 (v:v) 1 mM ammonium formate 0.5% FA (final volume: 200 μL; dilution: 8-fold).

### Preparation of Urine Samples

Spot urine was collected from 10 individuals and pooled together; urine was kept at −80 °C prior to analysis. Urine was thawed on ice and vortexed for 15 s before use. Sample aliquots (5 μL), SIL in MeOH (5 μL) and 190 μL of water 1 mM ammonium formate, and 0.5% FA were loaded into 96-well multifilter plates (Millipore), and samples were filtered using a positive-pressure 96-manifold (Water, Milan, Italy) for 5 min at 3 psi and collected in 350 μL 96-well plates.

### UHPLC-ESI-MS/MS Analysis

UHPLC-ESI-MS/MS was conducted on an AB Sciex 6500+ triple quadrupole coupled to a Shimadzu LC-30 AD pump (AB Sciex, Milan, Italy). Chromatographic separation was performed on an Acquity Premier HSST3 2.1 × 100 mm (1.8 μm particle size) purchased from Waters (Milan, Italy). Mobile phase A was water 0.1% FA; mobile phase B ACN 0.1% FA. The linear gradient, at a constant flow of 0.3 mL/min, started at 2% B and reached 5% at 0.5 min, 8% B at 1 min, 10% B at 1.5 min, 12.5% B at 2 min, 25% B at 3 min, 30% B at 4 min, 45% B at 5 min, 55% B at 6 min, 75% at 7 min, 85% B at 7.5 min, and then 98% B at 8 min. Final conditions were kept for 2 min, and then the column was re-equilibrated under initial conditions for 5 min. Total analysis time was 15 min. The column oven was set at 40 °C. *R*_0_ solvent was water 0.1% FA, and *R*_3_ solvent was 2-propanol. The injection volume was 5 μL for plasma and serum and 1 μL for urine samples.

ESI was operated in positive- and negative-ion modes. Two multiple reaction monitoring (MRM) transitions were set for each metabolite with a target cycle time across the MRM experiment set at 0.1 s. Curtain gas (CUR) was set at 35 PSI, and ion source (IS) gas 1 and 2 were set at 45 and 55 PSI respectively; CUR and IS were air, collision gas was nitrogen. Source temperature was 400 °C; ion spray voltage was 5500 V in positive ion mode and 4500 V in negative-ion mode. Compound-dependent parameters declustering potential (DP), entrance potential (EP), collision energy (CE), and collision-cell exit potential (CXP) were determined by direct infusion of the pure standard. Scheduled ionization was set from 0.5 to 8.5 min.

#### Data Processing

Data were processed using MultiQuant 3.0 software (AB Sciex, Milan, Italy). Statistics was performed with Statistica v.13.3 (TIBCO Software Inc., Palo Alto, CA, USA) and Metaboanalyst 5.0 (www.metaboanalyst.ca).

### Method Validation

#### Linearity and Limit of Quantitation (LOQ)

Calibration ranges were designed according to previous studies conducted in our laboratory on human plasma and urine,^56^ as well as from our experience of application in several clinical and nutritional studies, and from the ranges reported in the literature. Calibration standards were evaluated at 14 concentration levels, prepared by diluting stock solution in water:ACN 95:5 1 mM ammonium formate 0.5% FA for plasma and serum and water 1 mM ammonium formate and 0.5% FA for urine (Table S1). A linear polynomial model was employed with the 1/*X* weighing factor. A correlation coefficient (*r*^2^) greater than 0.990 was indicative of good linearity in a specific concentration range. The LOQ was calculated by determining the lowest calibration point with a signal-to-noise (S/N) of 10.

#### Matrix Effect

The matrix effect (ME) was evaluated using the matrix match calibration (MMC). For plasma and serum, calibration curves at 14 concentration levels were prepared by diluting stock solution in water:ACN 95:5 1 mM ammonium formate 0.5% FA (Solvent calibration, SC). Plasma and serum aliquots (25 μL) were extracted as described above, and dried samples were reconstituted in 200 μL of SC (MMC). For urine, SC was prepared by diluting stock solution in water 1 mM ammonium formate, 0.5% FA while MMC was prepared by adding 5 μL urine to 195 μL of SC. ME% was calculated as follows: (MMC slope MMS/SC slope) × 100. Acceptable ranges were 80–120% ME.

#### Recovery

Metabolite and SIL recoveries were evaluated at three different concentrations, low, medium, and high. The low and high concentrations were set as 4-fold lower and higher than the medium value. Analytical recovery was assessed using the postextraction standard addition method. For pre-extraction spiking (PRE-SP), standards in MeOH (25 μL) at three concentrations were spiked into both matrices (25 μL) prior to extraction, and samples were processed as described above. For postextraction standard addition (POST-SP), neat MeOH (25 μL) was added to blank plasma and serum (25 μL), and extracted matrices were reconstituted by adding 25 μL of standards in MeOH at low/medium or high concentration and 175 μL of water 1 mM ammonium formate 0.5% FA. Recovery was calculated as follows: % recovery = (PRE-SP concentration)/(POST-SP concentration) × 100. Seven replicates were analyzed for each concentration level. For urine, postextraction spiking was conducted after urine has been filtered.

#### Intra and Interday Precision and Accuracy

Intraday repeatability and accuracy were assessed by analyzing samples (*n* = 7) spiked at medium concentration. Interday repeatability and accuracy were assessed by analyzing the same samples at days 3 and 5. Precision is expressed as the coefficient of variation percentage (CV%); for acceptance, CV% is required to be lower than 15%. Accuracy is expressed as ratio (detected metabolite concentration/spiked concentration) × 100.

#### Metabolite Stability

Metabolite stability in aqueous solution was tested at time 0, 24, 48, and 72 h at 5 °C. Metabolites at medium concentration were spiked into 200 μL of water 1 mM ammonium formate 0.5% FA and divided into four vials that were let in an autosampler until analysis. Stability was calculated as (average concentration at time *X*/average concentration at time 0) × 100. Overall variation was expressed as CV% calculated considering all time points.

#### Phospholipid Removal and Carryover Effects

Phospholipid clean-up by hybrid-SPE was evaluated by performing a precursor ion scanning of *m/z* 184.3 (protonated phosphocholine) on extracted plasma and serum samples. For a direct comparison, plasma and serum aliquots (25 μL) were diluted in 175 μL of water:ACN 1 mM ammonium formate 0.5% FA. The carryover effect was monitored by injecting neat MeOH after the highest point of calibration curves and at specific intervals during the analytical sequence.

#### Analytical Performance during Method Validation

Analytical performance during method validation was evaluated by injecting a mix of SILs spiked at medium concentration in water 1 mM ammonium formate 0.5% FA throughout the analytical sequence.

##### Method Application to Biological Samples

This analysis was conducted as part of the European Joint Programming Initiative (JPI) “A Healthy Diet for a Healthy Life”, HEALTH through nutrition, microbiota and tryptophan bioMARKers (HEALTHMARK) project, aiming at studying the complex associations between GM, metabolic health, and the influence of gut metabolism on TRP and TYR metabolic pathways and diet.

#### MUCOL Study

The MUCOL study was a 12-week, randomized, double-blinded, placebo-controlled study to evaluate the efficacy of a probiotic on blood lipids and cholesterol levels in healthy mildly hypercholesterolaemic adults. The study was performed at the APC Microbiome Ireland and Atlantia Food Clinical Trials in Cork, Ireland. The study protocol was approved by the Cork Research Ethics Committee of the Cork Teaching hospitals. Healthy adult participants (*n* = 86) between the age of 20 and 70 years were recruited in Cork, Ireland and assessed for eligibility according to the following criteria: inclusion criteria included mild hypercholesterolemia as defined as a total cholesterol level between 5.5 and 8 mmol/L (measured by fasting blood sample at screening visit), BMI between 18.5 and 32 kg/m^2^, stable body weight (no more than 5% change) over the last 3 months, and otherwise general good health. Participants were excluded from the study if they were smokers, had any acute or chronic illnesses (including gastrointestinal disorders or surgery) and malignant or concomitant end-stage organ disease, were taking any medication (including cholesterol lowering medication) in the past month, were taking any probiotic and prebiotic supplements, had any blood transfusions in the last 6 months, taken any antibiotics in the last 3 months, had any history of drug or alcohol abuse at the time of enrolment, had an abnormal γ-GT, had made any major dietary changes in the last 3 months, and in case of female participants, they were pregnant, lactating, or wish to become pregnant during the study period, peri-, menopausal, or postmenopausal.

Study visits and sample collection occurred at baseline, week 4, week 8, and week 12 for study assessments. Fasting blood and urine samples were collected at each study visit, processed according to methods established in the laboratory, and stored at −80 °C until further analysis. The probiotic intervention involved administration of a *Lactobacillus mucosae* culture.

Quality control (QC) samples were created by pooling 25 μL of plasma or urine and then prepared as described above for plasma and urine independently. QC samples were injected 10 times prior to sample acquisition to condition UHPL and MS and then at fixed intervals throughout the sequence to evaluate analytical performance (*n* = 14). Dataset MTBLS4399 is available free of charge at ref (59).

##### Safety

Risks are associated with use of chemicals ACN, MeOH, and AF and use of use of pure chemical standards.

## Results and Discussion

The methodology described in this paper is designed for the accurate quantitation of 89 metabolites in three of the most used biological fluids in clinical metabolomics, human plasma, serum, and urine. To date, no one single analytical LC–MS/MS method covers such a large number of TRP, TYR, and PHE catabolites and other important metabolites. Our method is applicable to nutritional, clinical, and epidemiological studies with large cohorts, given its high-throughput and fast analysis time, and provides access to the fast quantitative analysis of dozens of neglected metabolites allowing to improve the investigation of the multiple correlations among these metabolic pathways.

Several human diseases have been linked to dysbiosis and altered TRP metabolism, such as IBD or IBS.^6−10^ Rapid and inexpensive urine test kits are used to reveal dysbiosis together with DNA analysis, but the former are not 100% reliable. These tests are based on colorimetric changes upon the presence of certain metabolites, as SKA or indoxyl-β-glucoside (plant indicant, PLI). PLI is a common urinary metabolite of TRP, and elevated levels are found in patients with Hartnup disease, where unabsorbed TRP is transformed into PLI by GM. Once exposed to air, PLI oxidizes and dimerizes, forming indigo dye responsible for the blue coloration of diapers in children affected by blue diaper syndrome. Our method enables accurate quantitation of such metabolites and, therefore, can provide useful information about the altered GM composition in humans, on normal or pathological metabolite concentration ranges, on the effect of intervention, and to better correlate metabolites and GI tract putatively affected by dysbiosis.

**Figure 1:**
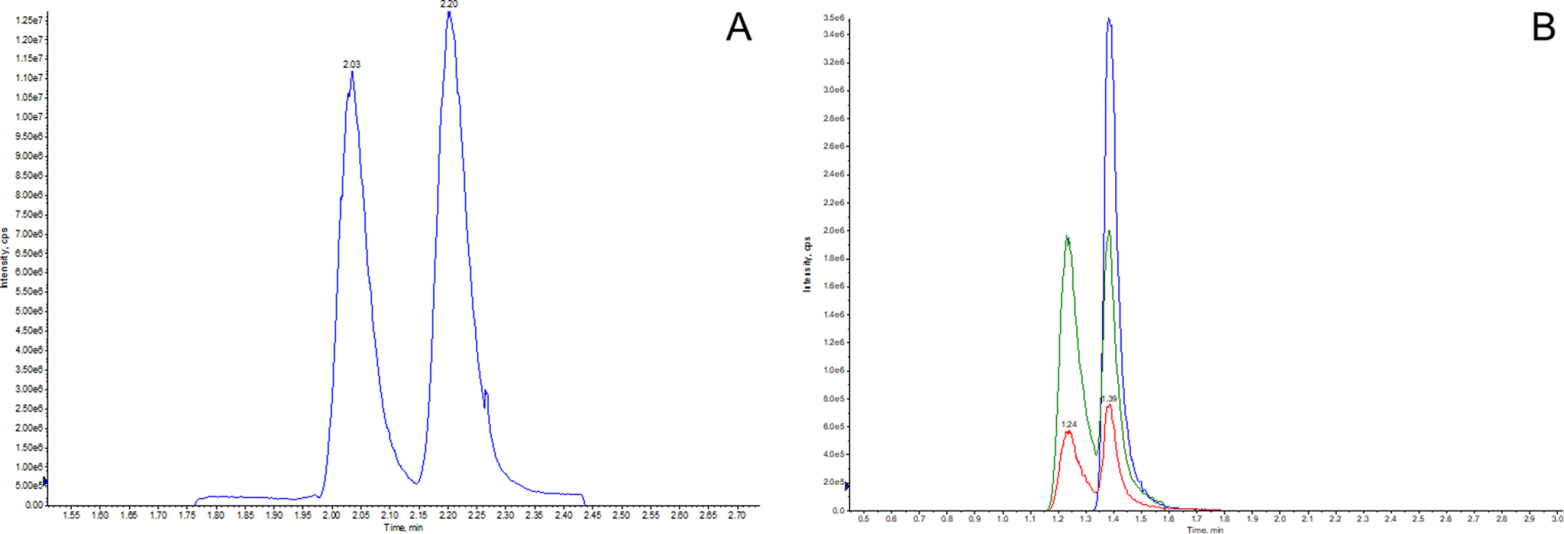
(A) Extracted ion chromatogram (XIC) of MRM (132.2 > 86.2) for detection and quantification of ILE (RT 2.03 min) and LEU (RT 2.20 min). (B) XIC of MRM (124.0 > 106.0; red line), (124.0 > 78.0; green line), and (124.0 > 80.0; blue line) specific for PA (RT 1.25 min) and PA (RT 1.38 min).

Several improvements were introduced compared to the method previously developed by our group.^56^ First, several new metabolites were introduced to maximize the coverage of the metabolic pathways of TRP, TYR, and PHE. Many of these are often neglected, but potentially important to interpret the perturbation of the pathways observed in clinical studies (PAGLY, PAGLU, PCS, PCG, IAG, and IBG). Other complementary metabolites were inserted due to their growing importance for human physiology and, because they are often required within the same clinical study, can be easily included within the method (TMA, TMAO, HSM, HSD...). Second, the column length was reduced from 150 to 100 mm, enabling faster chromatographic separation while retaining metabolite separation. Great effort was dedicated to the separation of most polar compounds and, in particular, to the separation of ILE from LEU and PA from NA. A slow gradient, starting from 98% of mobile phase A, was exploited to achieve this goal. This allowed the baseline separation of ILE and LEU, as demonstrated in Figure 1A, even better than what previously reported.^56^ Composition of reconstitution solvent after extraction of metabolites from plasma and serum was finely tuned, and the best results were obtained using water:ACN 95:5 (v:v) 1 mM ammonium formate, 0.5% FA. The concomitant addition of 0.5% FA and 1 mM ammonium formate enabled separation of PA from NA (Figure 1B). Higher concentrations of ammonium formate (10 mM) resulted in peak broadening. A small amount of ACN in reconstitution solvent (5%) helped the recovery of more hydrophilic compounds, as CNBA and TRPT. A higher percentage of ACN, greater than 15%, resulted in peak broadening of several hydrophilic compounds, mainly HSM, HSD, VAL, PA, NA, ME, and QA. Peaks eluting after 2 min are not affected by the percentage of ACN in reconstitution solvent. Other metabolites of TRP, TYR, and PHE catabolic pathways that are important for human physiology were tested but excluded from validation because of several reasons (Table S3).

### Analytical Specificity

Two unique MRM transitions were selected for each metabolite. MET-D_4_ (RT 1.48 min) and DA (RT 1.60 min) elute very close and share the most intense MRM (154.3 > 137.1). Interference from MET-D_4_ on DA quantitation was detected during method development and, therefore, this MRM was discarded in favor of two other MRM transitions listed in Table 1. Similarly to what is described in ref (55), PA and NA shared two MRM transitions (124.0 > 78) and (124.0 > 106.0); under our experimental setup NA can be quantified using MRM (124.0 > 80), which is not shared with PA (Figure 1B). Similarly, NAC-5HT and TRP ME share the same MRM (219.2 > 160.1) but metabolites are well chromatographically separated (RT 4.36 and 4.56 min, respectively). HIP was present in a wide concentration range in urine (0.45–200 ppm), and detector saturation was reached using MRM (180.2 > 105.0) for values greater than 100 ppm. To increase the linear range in urine analysis, MRM (180.2 > 77.1) was used as Q ions and MRM (180.2 > 51.0) was set as the q ion (Table 1). Given the great number of screened metabolites, it was not possible to obtain SILs for all compounds. The SIL number was increased from 7 to 15, enabling accurate quantitation of ILE, LEU, KYN, XA, IS, HIP, IAA, and CA. Analytical recoveries of other metabolites were corrected by basing (a) on closely eluting SILs (e.g., MET-D_4_ for VAL, PA, NA, and QA) or (b) on molecular similarity (e.g., KYN-D_4_ for 3OH-KYN or IAA-D_5_ for IAA ME and IAA EE).

### Extraction from Plasma and Serum by Hybrid-SPE

Hybrid-SPE technology allows fast sample clean-up and high throughput. Protein precipitation is achieved by adding precipitation agents, mainly ACN 1% FA or MeOH 1% ammonium formate. After vigorous shaking, proteins are precipitated at the bottom of each well and are retained by a membrane, while the sample diluted in solvent is filtered and recovered in a plate. Phospholipid (PL) removal is achieved by Lewis acid–base interaction between negative charge of PL heads and zirconia or tungsten atoms incorporation into the well. According to the manufacturer’s procedure, the minimum required sample volume is 50 μL; in this work we were able to successfully obtain quantitative data by loading 25 μL and by diluting in equal amounts of MeOH containing SILs. Lowering sample loading reduces ACN volume required for extraction (150 μL in total vs 300 μL in standard procedure) and, therefore, reduces evaporation time (less than 1 h at 37 °C and nitrogen flow at 1.5 psi). ACN has to be preferred over MeOH as it (a) is more volatile and (b) reduces sample contamination by PL during the second extraction step. In fact, MeOH is able to desorb a notable amount of lyso-PC and PC during the second extraction step compared to ACN (Figure S5).

We also tested the minimum number of steps required to achieve satisfactory recovery. Up to 90% of metabolites are recovered by performing a two-step extraction. Compared to ref (56), each shaking step was reduced from 10 to 5 min, minimizing any potential loss of sample and reducing overall extraction time. A further improvement was the introduction of a two-step sample reconstitution after evaporation that enabled an overall metabolite recovery. In fact, washing plate wells with MeOH demonstrated that metabolites, especially those present at higher concentrations, were not completely recovered by a single reconstitution. Performing an extra washing with reconstitution solvent increased overall metabolite recovery (Figure S6).

### Linearity and LOQ

Human biofluids contain metabolite present in different concentration ranges, from ppb to ppm, and deep differences are detected between plasma/serum and urine. Therefore, calibration curves were specifically designed to cover expected concentration in different biofluids based on prior knowledge^56^ and by preliminary analysis of real biological samples. For metabolites not detected in human biofluids, the lower range of concentrations was selected. Metabolites present at high concentrations are linear across four orders of magnitude, from ppm to ppb. For example, TRP in plasma is linear from 0.003125 to 3.2 ppm (Table S4). An eightfold dilution of plasma and serum and a 40-fold dilution of urine enabled the quantification of low abundant metabolites down to few ppb and, at the same time, prevented detector saturation from high abundant ones. Exceptions were IND and SKA in positive ion mode whose LOQs were 0.4 and 0.2 ppm in plasma/serum and 0.8 and 0.4 ppm in urine respectively, and HVA and DOPAC in negative-ion mode, whose LOQs were 0.05 and 0.025 ppm in plasma/serum and 0.1 ppm in urine, respectively.

### Matrix Effect

The ME was in acceptable ranges (80–120%) for all metabolites in plasma and serum samples except for GABA, EPI and IND in serum (Table S4). Similarly, GABA was strongly affected in urine (13.7%) together with HSD (39.2%). GABA and HSD are practically unretained under our chromatographic setup, and this could be the reason for such deviation. Therefore, we propose to use data on GABA in all matrices and HSD in urine to detect fold changes.

### Recovery

Analytical recovery in plasma and serum was generally greater than 80% with some exceptions, such as BA (75.3%), IACR (73.4%), and CNBA (78%) in serum at spiked low concentration, TRPT (78.4) and IAA ME (77.1%) in serum spiked at medium concentration, or BA (70.3%), KYN-D_4_ (71.5%), and 5ME-IAA (75.3%) in plasma spiked at low and medium concentrations, respectively (Table S5). HST recovery in plasma spiked at three concentrations was lower than 80%. Similarly, QA was slightly under 80% in all matrices; low recoveries of dicarboxylic acid can be experienced in hybrid-SPE when using ACN 1% FA as a precipitating agent.^60,61^ IND and SKA were poorly recovered from plasma and serum probably because of evaporation; in fact, a clear smell of IND and SKA was perceived when drying samples down. Direct injection of the hybrid-SPE precipitate can be performed to quantitative analyze IND and SKA. Several attempts were made to avoid evaporation/reconstitution steps but results were not satisfactory, especially for the chromatographic separation and peak shapes of the most polar metabolites.

KA, KA-D_5_, XA, and XA-D_4_ recoveries were all slightly lower than 80% in both matrices; a possible explanation is the low solubility of KA and XA in water. The reconstitution solvent composition can affect the overall metabolome coverage.^62−65^ Given the diverse chemical and physical properties of metabolites covered in this work, several experiments were conducted to find the optimal reconstitution solvent, capable of achieving high metabolite recoveries and maintaining acceptable chromatographic separations. In this sense, the addition of MeOH or DMSO did not increase the overall recovery of XA and KA.

Dilution and the shot technique used for urine analysis guaranteed good accuracy. The exception was NOR, whose recoveries were 70.8% at low and 66% at high concentrations, respectively.

### Precision and Accuracy

Intraday accuracy was good for most of the metabolites, errors being lower than ±10% in all biofluids (Table S6). Exceptions were, in plasma: 5OH-TRP, XA-D_4_, HVA at day 1, IGLUT at days 1 and 3; in serum: IGLUT and BA at day 1,6-SME day 3, 4OH-PPA and IACR at days 1 and 3; in urine: 5ME-IET at day 1, 2 AM at day 3, and SKA at day 5. Interday CV% were below 15% for all metabolites except for NOR in urine.

Intra and interday precisions were satisfactory (CV% < 15%) for most of the metabolites (Table S7). Exception was NOR at DAY 5 in plasma (17.0% CV).

### Metabolite Stability

After 24 h at 5 °C in the autosampler, the majority of metabolites showed stability greater than 90%, enabling accurate quantitation of a 96-well plate (Table S7). Exceptions were TRTP (87%) and 5OH-IET (87%). At 48 h, TRP ME (89.2%), IACR (87.5), KYN-D_5_ (86.1%), TRPT (79.0%), TROL (82.8%), and 5OH-IET (76.6%) were below 90% stability. After 72 h, KYN-D_5_ (89.9%), NAC-TRP EE (89.8%), IAA ME (89.3%), IAA EE (89.2%), TROL (87.3%), PPG (86.3%), 4OH-PAG (84.5%), IND (88.6), IACR (79.9%), 5OH-IET (79.5%), and TRPT (74.9%) were below 90%. The marked decrease of TRPT in aqueous solution could be the result of its poor solubility and stability in aqueous solution. To minimize this variation, action must be taken prior to the analytical run. In particular, in agreement with good practice in nutritional metabolomics, a systematic scheme for the sample run order should be created, by combining the experimental design with complete or groupwise randomization.^66^ The complete randomization is the preferred solution, but also the partial randomization is acceptable in case of a large number of samples to be analyzed.

### Sample Clean-up

Hybrid-SPE technology enabled complete removal of PL from plasma and serum samples, as also reported in ref (56). Appropriate sample clean-up reduced ME and source contamination while increasing column life span.

### Carryover

No metabolite carryover was detected within run, even after injection of the highest point of the calibration curve or after seven samples spiked at high metabolite concentration, as shown in Figure S7.

### Analytical Performance during Method Validation

CV% for SILs in QC injected at fixed intervals was below 15%, indicating good analytical performance throughout all acquisition sequences (Table S8).

### Method Application to Biological Samples

The method was tested on plasma and urine samples obtained from the same individuals in the frame of the MUCOL study.

**Figure 2:**
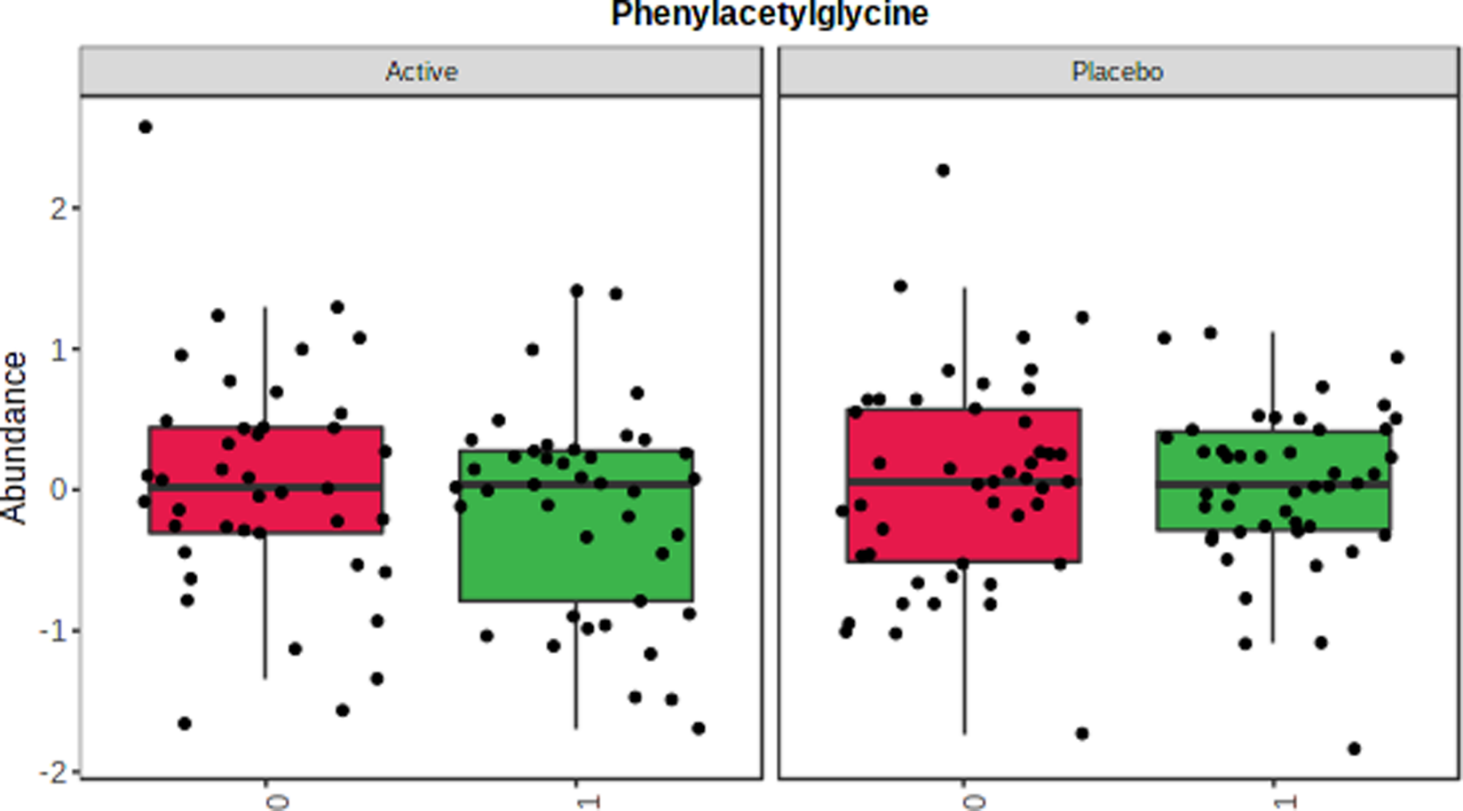
Graphical summary of PAGLY distribution in active and placebo groups before (red) and after (green) intervention. Data were log-transformed and Pareto-scaled. Leverage: 0.081461; SPE: 8.38E^–32^.

The concomitant analysis of plasma (*n =* 319) and urine (*n =* 298) from the same individual allows drawing important information on host-GM cometabolism. A total of 51 metabolites were detected and quantified, with concentrations spanning from nM to mM (Table 2). Among them, five were exclusively found in plasma (IAA ME, IPA, IBA, ICARB, and PLA), 17 were quantified in urine (NOR, EPI, DA, 3ME-TYRA, TYRA, HVA, HVAS, NA, PA, PAG, 4OH-PAG, PPG, IBG, NAC-TRP, 5OH-TRP, IGLUT, and NAC-TYR), and 30 were found in both biofluids (MET, VAL, ILE, LEU, TMA, TMAO, HSM, HSD, TRP, KYN, 3OH-KYN, KA, XA, 3OH-AA, QA, 5-HT, 5OH-IAA, IAG, IS, IAA, ILA, PHE, NAC-PHE, TYR, PCS, PCG, 4OH-PLA, CYG, PAGLU, and HIP) (Figure 2). Notably, DA and PA in plasma were under the quantitation limit while PLI was below the quantitation limit in urine. It is interesting to note that four microbial metabolites derived from TRP (IAA ME, IPA, IBA, and ICARB) and one derived from PHE catabolism (PLA) are not excreted in urine; this might indicate a relevant role of these metabolites for human health. For example, IPA is known to be an antioxidant^36,40^ while ICARB has been recently shown to be diminished in obese subjects compared to nonobese ones.^11^ Little is known about IAA ME and IBA effects on human health. Most end point catabolites of TRP, TYR, and PHE were detected in urine, such as IS, IBG, 4OH-PPG, PCS, PCG, HVAS, CYG, PPG, HIPP, PAGLY, and PAGLU. Notably, PAGLU has been detected in both biofluids while PAGLY only in urine.

**Table 2:** Metabolites Quantified in Plasma and Urine of the MUCOL Study^a^

name	plasma (μM)			urine (μM)		
	min	median	max	min	median	max
histamine	0.02	0.05	0.23	0.28	1.15	4.75
histidine	0.36	1.49	8.43	7.05	107.81	508.91
TMA	0.99	1.64	4.48	0.07	9.14	35.65
TMAO	0.25	1.49	28.66	27.20	1219.84	9711.94
norepinephrine	u.d.l.			1.27	15.38	66.33
epinephrine	u.d.l.			0.61	1.21	2.19
l-valine	27.82	65.61	202.38	0.24	34.23	195.99
picolinic acid	u.d.l.			0.0009	0.54	4.58
nicotinic acid	u.d.l.			0.01	0.69	3.66
l-methionine	2.84	11.02	37.91	0.58	8.21	41.23
quinolinic acid	0.53	1.08	6.80	1.52	34.00	173.49
dopamine	u.d.l.			0.002	0.80	2.78
3-hydroxykynurenine	0.03	0.21	0.86	0.03	1.12	11.06
l-isoleucine	10.79	43.55	182.15	0.02	9.57	99.98
l-tyrosine	24.39	55.47	93.55	0.06	25.94	153.93
tyramine	u.d.l.			0.01	1.53	7.34
l-leucine	33.75	78.24	180.89	0.06	25.94	153.93
serotonin	0.003	0.01	1.20	0.18	2.69	14.36
3-methoxy-tyramine	u.d.l.			0.01	0.21	3.85
5-hydroxy-l-tryptophan	u.d.l.			0.80	5.57	24.14
l-kynurenine	0.07	0.21	0.86	0.29	2.08	27.73
l-phenylalanine	0.18	34.54	69.44	2.16	49.46	336.70
3-hydroxyanthranilic acid	0.01	0.07	5.97	0.33	6.06	44.09
l-tryptophan	22.48	51.93	96.79	5.47	77.59	468.57
xanthurenic acid	0.07	0.19	0.33	0.08	1.84	10.47
4-hydroxyphenyl-lalctic acid	0.35	0.94	2.94	0.02	5.41	78.54
homovanillic acid sulfate	u.d.l.			1.74	49.88	284.36
*N*-acetyl-l-tyrosine	u.d.l.			0.04	0.84	6.77
kynurenic acid	0.00003	0.01	0.11	0.95	2.08	27.73
4-hydroxyphenylpropionylglycine	u.d.l.			0.00008	0.02	1.13
5-hydroxyindole-3-acetid acid	0.02	0.06	0.72	2.51	28.59	472.54
indoxyl-*β*-glucuronide	u.d.l.			0.39	7.53	85.80
indoxyl sulfate	0.53	3.68	11.81	29.44	485.25	3144.40
phenylacetyl-l-glutamine	0.19	2.62	16.61	97.14	863.33	3354.59
hippuric acid	0.11	2.56	57.50	100.78	1867.39	60983.60
homovanillic acid	u.d.l.			0.18	28.02	104.64
phenylacetylglycine	u.d.l.			0.08	1.79	119.24
*p*-cresol glucuronide	0.00007	0.03	0.51	2.60	72.37	672.78
*p*-cresol sulfate	0.37	22.71	101.68	58.83	1425.82	9646.17
*N*-acetyl-l-phenylalanine	0.00003	0.00238	0.06	0.14	1.30	8.34
phenyllactic acid	0.01	0.15	0.53	u.d.l.		
*N*-acetyl-l-tryptophan	0.0014	0.56	7.27	0.0014	0.56	7.27
indole-3-lactic acid	0.15	0.55	3.21	0.01	1.23	34.55
phenylpropionylglycine	u.d.l.			0.0036	0.20	4.69
indole-3-acryloylglycine	0.002	0.02	0.15	1.66	66.54	1322.32
cinnamoylglycine	0.0005	0.17	1.32	0.49	33.75	447.84
indole-3-carboxaldehyde	0.02	0.05	0.14	u.d.l.		
indole-3-acetic acid	0.35	1.13	10.69	0.47	11.14	194.34
indole-3-propionic acid	0.003	0.86	7.05	u.d.l.		
indole-3-butyric acid	0.0001	0.01	0.03	u.d.l.		
indole-3-acetic acid, methyl ester	0.00058	0.01	0.04	u.d.l.		

a Data are expressed as μM.; u.d.l.: under the detection limit.

**Figure 3:**
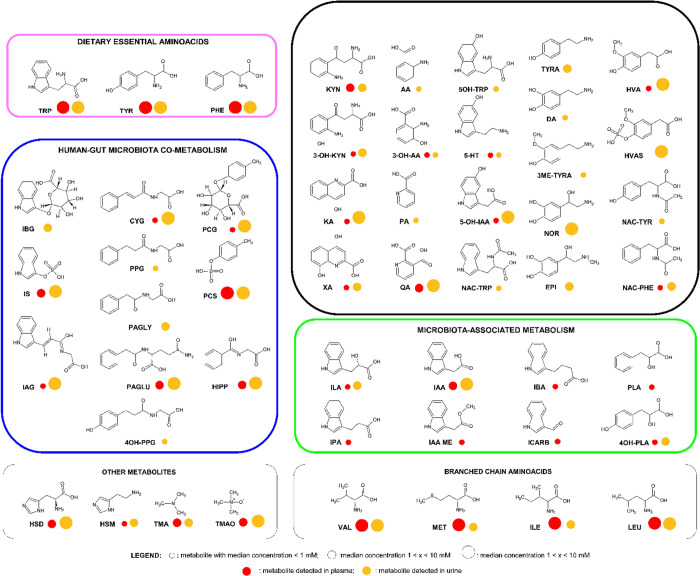
Schematic representation of 52 metabolites detected in plasma (red circle) and urine (yellow circle) samples of the MUCOL study.

QC analysis revealed good system stability for both matrices, CV% being lower than 15% for all SILs, except for DOPAC-D_5_ in urine (CV 25.5%) (Table S9). After performing statistical analysis, some metabolites were significantly changed in the intervention group. ASCA, a multivariate extension of analysis of variance that considers time per treatment interaction, revealed that, for example, PAGLY was found to be diminished in the probiotic group compared to placebo, as can be seen in Figure 3. The complete result dissemination and discussion will be the subject of a separate paper.

## Conclusions

A high-throughput UHPLC-ESI-MS/MS method was validated for the simultaneous quantitation of up to 89 metabolites in human plasma, serum, and urine. Most of the metabolites derived from catabolism of essential amino acids TRP, TYR, and PHE operated by the host and GM, while others were included because of their important roles in human physiology. Plasma and serum samples were loaded on 96-well plates for protein precipitation and phospholipid removal by hybrid-SPE technology. Urine was 40-fold diluted and filtered. The method was designed for low sample volume requirement and fast and minimum processing while ensuring satisfactory metabolite recoveries, minimal ME, and high accuracy and precision. A total analysis time of 15 min enables analysis of 96 samples per day. The validated method was tested on plasma and urine samples obtained from the same individual in the frame of the MUCOL study, allowing to precisely quantify 51 metabolites and providing important information on the metabolic fate of essential amino acids and on their host-gut-microbiota cometabolism. Our method is applicable to large cohorts, including observational and epidemiological studies, where thousands of samples can be processed and accurate results must be obtained in a reasonable amount of time and with low operating costs. The method could be considered the reference for understanding changes in human-gut microbial cometabolism in health and disease.

## Supporting Information Available

Table S1: information on the accession number of tested metabolite in public repositories (HMDB and PubChem) and quantitation ranges used in plasma, serum, and urine analysis; Table S2: details of SIL spiked and final concentrations in plasma, serum, and urine; Table S3: metabolites excluded from further validation and technical reasons; Table S4: information on the linear range, coefficient of determination (*R*^2^), and matrix effect in plasma, serum, and urine; Table S5: the percentage of recovery at three spiked concentrations in plasma, serum, and urine; Table S6: intraday analytical accuracy; Table S7: intraday and interday analytical precision; Table S8: details about metabolite stability in water; Table S9: CV% for QC analysis during method validation; Table S10: % CV for plasma and urine QC analysis; Figure S1: TRP catabolic pathways through KYN and hydroxylation pathways; Figure S2: microbiota-associated tryptophan catabolic pathways; Figure S3: TYR metabolism; Figure S4: PHE metabolism; Figure S5: the effect of using ACN and methanol during extraction with hybrid-SPE; Figure S6: the effect of performing one or two reconstitution steps; and Figure S7: optimal needle cleaning and no carryover effect observed after injection of samples spiked at high metabolite concentration (PDF)
