# Supplementary material for: Metabolomic Workflow for the Accurate and High-Throughput Exploration of the Pathways of Tryptophan, Tyrosine, Phenylalanine, and Branched-Chain Amino Acids in Human Biofluids
Source: J Proteome Res. 2022 Apr 5;21(5):1262–75. doi: 10.1021/acs.jproteome.1c00946 (PMC9087329; doi:10.1021/acs.jproteome.1c00946)
Supplement: Supplementary file 1 — pr1c00946_si_001.pdf [file pr1c00946_si_001.pdf]

Supporting Information for:

A metabolomic workflow for the accurate and high-throughput exploration of the pathways of tryptophan, tyrosine, phenylalanine and branched chain amino acids in human biofluids.

Andrea Anesi [1], Kirsten Berding[2], Gerard Clarke [2,3], Catherine Stanton[2], John F. Cryan[2,4], Noel Caplice [2,5], Paul Ross[2], Andrea Doolan[6], Urska Vrhovsek [1], Fulvio Mattivi [1,7] *

* Corresponding author

[1] Unit of Metabolomics, Department of Food Quality and Nutrition, Research and Innovation Centre, Fondazione Edmund Mach (FEM), San Michele all'Adige, Italy

[2] APC Microbiome Ireland, University College Cork, Ireland

[3] Department of Psychiatry and Neurobehavioural Sciences, University College Cork, Cork, Ireland

[4] Department of Anatomy and Neuroscience, University College Cork, Cork, Ireland

[5] Centre for Research in Vascular Biology, University College Cork, Cork, Ireland

[6] Atlantia Food Clinical Trial, Blackpool, Cork, Ireland

[7] University of Trento, Department of Cellular, Computational and Integrative Biology (CIBIO), Trento, Italy

Supporting information contains eight tables: **Table S1** provides information on accession number of tested metabolite in public repositories (HMDB and PubChem) and quantitation ranges used in plasma, serum and urine analysis; **Table S2** provides details on SIL spiked and final concentrations in plasma, serum and urine; **Table S3** reports metabolites excluded from further validation and technical reasons; **Table S4** provides information on linear range, coefficient of determination (R^2) and matrix effect in plasma, serum and urine; **Table S5** presents the percentage of recovery at three spiked concentration in plasma, serum and urine; **Table S6** details intra-day analytical accuracy; **Table S7** reports intra-day and inter-day analytical precision; **Table S8** provides details about metabolite stability in water; **Table S9** reports CV% for QC analysis during method validation; **Table S10** reports % CV for plasma and urine QC analysis.

Supporting information contains seven figures: **Figure S1** illustrates tryptophan catabolic pathways through kynurenine and the hydroxylation pathway; **Figure S2** depicts microbiota associated tryptophan catabolic pathways; **Figure S3** illustrates tyrosine metabolism; **Figure S4** illustrates phenylalanine metabolism; **Figure S5** shows the effect of using acetonitrile and methanol during extraction with Hybrid-SPE; **Figure S6** reports the effect of performing one or two reconstitution steps; **Figure S7** illustrated optimal needle cleaning and no carryover effect observed after injection of samples spiked at high metabolite concentration.

Supporting Table 1. Human Metabolome DataBase (HMDB) or PubChem accession numbers, working concentration ranges for plasma/serum and urine calibration curves.

Name	Abbreviation	HMDB PubChem ID	PLASMA/SERUM	URINE
			P1-P14 (ppm)	P1-P14 (ppm)
Histamine	HSM	HMDB0000870	1.6-0.00019532	1.6-0.00019532
Histidine	HSD	HMDB0000177	3.2-0.00039063	6.4-0.00078125
Trimethylamine	TMA	HMDB0000906	3.2-0.00039063	1.6-0.00019532
γ-aminobutyric acid	GABA	HMDB0000112	1.6-0.00019532	1.6-0.00019532
Trimethylamine-*N*-oxide	TMAO	HMDB0000925	3.2-0.00039063	25.6-0.003125
Norepinephrine	NOR	HMDB0000216	3.2-0.00039063	3.2-0.00039063
Epinephrine	EPI	HMDB0000068	1.6-0.00019532	1.6-0.00019532
L-valine	VAL	HMDB0000883	25.6-0.003125	3.2-0.00039063
Picolinic acid	PA	HMDB0002243	1.6-0.00019532	1.6-0.00019532
Nicotinic acid	NA	HMDB0001488	1.6-0.00019532	1.6-0.00019532
L-methionine-D_4	MET- D_4		6.4-0.00078125	1.6-0.00019532
L-methionine	MET	HMDB0000696	12.8-0.0015625	3.2-0.00039063
Quinolinic acid	QA	HMDB0000232	3.2-0.00039063	6.4-0.00078125
L-Dopa	L-DOPA	HMDB0000181	1.6-0.00019532	1.6-0.00019532
2-aminophenol	2AM	CID 5801	6.4-0.00078125	1.6-0.00019532
Dopamine- D_4	DA-D_4		0.8-0.000098	0.000098
Dopamine	DA	HMDB0000073	0.8-0.000098	0.000098
3-hydroxykynurenine	3OH-KYN	HMDB0011631	1.6-0.00019532	1.6-0.00019532
L-isoleucine-D_{10}	ILE-D_{10}		12.8-0.0015625	1.6-0.00019532
L-isoleucine	ILE	HMDB0000172	25.6-0.003125	3.2-0.00039063
L-tyrosine-D_4	TYR-D_4		12.8-0.0015625	3.2-0.00039063
L-tyrosine	TYR	HMDB0000158	25.6-0.003125	6.4-0.00078125
Tyramine	TYRA	HMDB0000306	0.8-0.000098	1.6-0.00019532
L-leucine-D_{10}	LEU-D_{10}		12.8-0.0015625	1.6-0.00019532
L-leucine	LEU	HMDB0000687	25.6-0.003125	3.2-0.00039063
Serotonin-D_4	5-HT-D_4		0.8-0.000098	1.6-0.00019532
Serotonin	5-HT	HMDB0000259	0.8-0.000098	1.6-0.00019532
3-methoxy-*p*-tyramine	3ME-TYRA	HMDB0000022	1.6-0.00019532	0.000098
5-hydroxy-L-tryptophan	5OH-TRP	HMDB0000472	3.2-0.00039063	1.6-0.00019532
N-methylserotonin	ME-5HT	HMDB0004369	1.6-0.00019532	1.6-0.00019532

L-kynurenine-D_4	KYN-D_4		1.6-0.00019532	1.6-0.00019532
L-kynurenine	KYN	HMDB0000684	1.6-0.00019532	1.6-0.00019532
L-phenylalanine	PHE	HMDB0000159	25.6-0.003125	6.4-0.00078125
4-hydroxyphenylacetylglycine	4OH-PAG	HMDB0000735	3.2-0.00039063	1.6-0.00019532
3-hydroxyanthranilic acid	3OH-AA	HMDB0001476	3.2-0.00039063	1.6-0.00019532
L-tryptophan-D_5	TRP-D_5		12.8-0.0015625	6.4-0.00078125
Xanthurenic acid-D_4	XA-D_4		0.8-0.000098	1.6-0.00019532
L-tryptophan	TRP	HMDB0000929	25.6-0.003125	6.4-0.00078125
Xanthurenic acid	XA	HMDB0000881	0.8-0.000098	1.6-0.00019532
3,4-dihydroxyphenylacetic acid-D_5	DOPAC-D_5		25.6-0.003125	12.8-0.0015625
3,4-dihydroxyphenylacetic acid	DOPAC	HMDB0001336	51.2-0.00625	12.8-0.0015625
3-(4-hydroxyphenyl)-lactic acid	4OH-PLA	HMDB0000755	3.2-0.00039063	3.2-0.00039063
Homovanillic acid sulfate	HVAS	HMDB0011719	6.4-0.00078125	6.4-0.00078125
N-acetyl-L-tyrosine	NAC-TYR	HMDB0000866	1.6-0.00019532	1.6-0.00019532
L-tryptophanol	TROL	HMDB0003447	0.8-0.000098	1.6-0.00019532
Kynurenic acid-D_5	KA-D_5		0.8-0.000098	1.6-0.00019532
Kynurenic acid	KA	HMDB0000715	0.8-0.000098	3.2-0.00039063
5-methoxy-L-tryptophan	5ME-TRP	HMDB0002339	0.8-0.000098	1.6-0.00019532
Tryptamine	TRYT	HMDB0000303	0.8-0.000098	1.6-0.00019532
4-hydroxyphenylpropionylglycine	4OH-PPG	HMDB0094725	1.6-0.00019532	1.6-0.00019532
5-hydroxytryptophol	5OH-IET	HMDB0001855	0.8-0.000098	1.6-0.00019532
5-methoxytryptamine	5ME-TRYT	HMDB0004095	1.6-0.00019532	1.6-0.00019532
6-sulfathoxymelatonin	6-SMEL	HMDB0041815	0.8-0.000098	1.6-0.00019532
5-hydroxyindole-3-acetid acid-D_5	5OH-IAA-D_5		0.8-0.000098	3.2-0.00039063
5-hydroxyindole-3-acetid acid	5OH-IAA	HMDB0000763	0.8-0.000098	6.4-0.00078125
Indoxyl-β-glucoside	PLI	HMDB0061755	1.6-0.00019532	3.2-0.00039063
N-acetyl-5-hydroxytryptamine	NAC-5HT	HMDB0001238	0.8-0.000098	1.6-0.00019532
Indoxyl-β-glucuronide	IBG	HMDB0010319	1.6-0.00019532	3.2-0.00039063
Indoxyl sulfate-D_4	IS-D_4		3.2-0.00039063	6.4-0.00078125
Indoxyl sulfate	IS	HMDB0000682	6.4-0.00078125	51.2-0.00625
Phenylacetyl-L-glutamine	PAGLU	HMDB0006344	1.6-0.00019532	51.2-0.00625
Hippuric acid-D_5	HIP-D_5		3.2-0.00039063	204.8-0.025
Hippuric acid	HIP	HMDB0000714	6.4-0.00078125	6.4-0.00078125

L-tryptophan, methyl ester	TRP ME	CID 77980	0.8-0.000098	1.6-0.00019532
Homovanillic acid	HVA	HMDB0000118	12.8-0.0015625	6.4-0.00078125
Phenylacetylglycine	PAGLY	HMDB0000821	1.6-0.00019532	3.2-0.00039063
Indole-3-acetyl aspartic acid	IASP	HMDB0038666	1.6-0.00019532	1.6-0.00019532
3-(4-hydroxyphenyl)-propionic acid	4OH-PPA	HMDB0002199	204.8-0.025	204.8-0.025
p-cresol glucuronide	PCG	HMDB0011686	1.6-0.00019532	12.8-0.0015625
Indole-3-acetamide	IACT	HMDB0029739	0.8-0.000098	1.6-0.00019532
L-tryptophan, ethyl ester	TRP EE	CID 81996	0.8-0.000098	1.6-0.00019532
4-hydroxycinnamic acid	HCA	HMDB0030677	1.6-0.00019532	1.6-0.00019532
p-cresol sulfate	PCS	HMDB0011635	12.8-0.0015625	51.2-0.00625
Indole-3-acetylglutamic acid	IGLUT	HMDB0038665	1.6-0.00019532	0.00039063
Anthranilic acid	AA	HMDB0001123	1.6-0.00019532	1.6-0.00019532
N-acetyl-L-phenylalanine	NAC-PHE	HMDB0000512	1.6-0.00019532	1.6-0.00019532
Phenyllactic acid	PLA	HMDB0000779	3.2-0.00039063	3.2-0.00039063
N-acetyl-L-tryptophan	NAC-TRP	HMDB0013713	1.6-0.00019532	1.6-0.00019532
Indole-3-lactic acid	ILA	HMDB0000671	1.6-0.00019532	3.2-0.00039063
N-acetyl-L-tyrosine, ethyl ester	NAC-TYR EE	CID 13289	1.6-0.00019532	1.6-0.00019532
Phenylpropionylglycine	PPG	HMDB0000764	1.6-0.00019532	1.6-0.00019532
Indole-3-acryloylglycine	IAG	HMDB0006005	1.6-0.00019532	25.6-0.003125
Indole-3-carboxylic acid	ICA	HMDB0003320	0.8-0.000098	1.6-0.00019532
Cinnamoylglycine	CYG	HMDB0011621	1.6-0.00019532	12.8-0.0015625
Indole-3-carboxaldehyde	ICARB	HMDB0029737	0.8-0.000098	1.6-0.00019532
Melatonin	MEL	HMDB0001389	0.8-0.000098	1.6-0.00019532
5-methoxytryptophol	5ME-IET	HMDB0001896	0.8-0.000098	1.6-0.00019532
5-methoxyindole-3-acetic acid	5ME-IAA	HMDB0004096	0.8-0.000098	1.6-0.00019532
Benzoic acid	BA	HMDB0001870	204.8-0.0125	204.8-0.0125
Indole-3-acetic acid-D_5	IAA-D_5		1.6-0.00019532	3.2-0.00039063
Indole-3-acetic acid	IAA	HMDB0000197	6.4-0.00078125	6.4-0.00078125
Indole-3-ethanol	IET	HMDB0003447	0.8-0.000098	1.6-0.00019532
Cinnabarinic acid	CNBA	HMDB0004078	1.6-0.00019532	1.6-0.00019532
Indole-3-acrylic acid	IACR	HMDB0000734	3.2-0.00039063	3.2-0.00039063
Indole-3-propionic acid	IPA	HMDB0002302	0.8-0.000098	1.6-0.00019532
trans-cinnamic acid-D_5	CA-D_5		3.2-0.00039063	1.6-0.00019532

trans-cinnamic acid	CA	HMDB0000930	3.2-0.00039063	1.6-0.00019532
N-acetyl-L-tryptophan, ethyl ester	NAC-TRP EE	CID 2724382	1.6-0.00019532	1.6-0.00019532
Indole-3-butyric acid	IBA	HMDB0002096	1.6-0.00019532	6.4-0.00078125
Indole-3-acetonitrile	IACN	HMDB0006524	0.8-0.000098	1.6-0.00019532
Indole-3-acetic acid, methyl ester	(IAA ME	HMDB0029738	0.8-0.000098	1.6-0.00019532
Indole	IND	HMDB0000738	51.2-0.00625	12.8-0.0015625
Indole-3-acetic acid, ethyl ester	IAA EE	CID 13067	0.8-0.000098	1.6-0.00019532
Tryptanthrin	TRPT	CID 73549	0.8-0.000098	1.6-0.00019532
3-methylindole	SKA	HMDB0000466	51.2-0.00625	12.8-0.0015625

Supporting Table 2. Working concentration of SIL spiked into plasma, serum and urine and their final concentration. SIL final concentration in plasma and serum is calculated considering an eight-fold dilution after re-constitution of dried samples. For urine, spiked concentrations already considers the dilution in final volume and, therefore, is the actual final concentration.

Name	PLASMA/SERUM		URINE
	Spiked concentration (ppm)	Final concentration (ppm)	Spiked concentration (ppm)
MET-D_4	1.6	0.2	0.05
DA-D_4	0.08	0.01	0.05
ILE-D_{10}	4	0.5	0.05
TYR-D_4	4	0.5	0.2
LEU-D_{10}	4	0.5	0.1
5-HT-D_4	0.08	0.01	0.02
KYN-D_4	0.04	0.005	0.01
TRP-D_5	4	0.5	0.5
XA-D_4	0.016	0.002	0.02
DOPAC-D_5	4	0.5	0.1
KA-D_5	0.016	0.002	0.1
5OH-IAA-D_5	0.04	0.005	0.1
IS-D_4	0.8	0.1	0.5
HIP-D_5	0.8	0.1	1
IAA-D_5	0.08	0.01	0.2
CA-D_5	0.8	0.1	0.01

Supporting Table 3. Metabolites excluded from validation and technical reasons.

Name	PLASMA
Indole-3-pyruvic acid	Metabolite instability in aqueous solution
Indole-3-glycoxylic acid	Metabolite instability in aqueous solution
Indole-3-methanol	1) metabolite instability in aqueous solution<br>2) in source loss of H_2O occurring, yielding *m/z* 130.0 ion common with all indoles and impossibility to get specific MRM transition due to co-elution with other indoles
3,3'-diindoylmethane	Poor recovery due probably to low solubility in aqueous solution
Phenylpropionic acid	Unsatisfactory results
3-(4-hydroxyphenyl)-acetic acid	Unsatisfactory results
6-hydroxymelatonin	Metabolite instability in aqueous solution
Indirubin	Low solubility in aqueous solution and aggregation causing clotting
Indigotin	Low solubility in aqueous solution and aggregation causing clotting

Supporting Table 4. Range of linearity (ppm), coefficient of determination (R^2) and matrix effect percentage (ME%) respect to solvent calibration for screened metabolites in plasma, serum and urine

Name (abbreviation)	PLASMA			SERUM			URINE		
	Linear range (ppm)	R^2	ME (%)	Linear range (ppm)	R^2	ME (%)	Linear range (ppm)	R^2	ME (%)
Histamine	1.6-0.000391	0.9951	101.0	1.6-0.000391	0.9940	101.9	0.8-0.003125	0.9991	91.1
Histidine	1.6-0.025	0.9995	97.2	1.6-0.025	0.9996	97.9	3.2-0.4	0.9986	39.2
TMA	0.2-0.001563	0.9958	108.8	0.2-0.001563	0.9992	118.6	0.4-0.00156	0.9919	93.5
GABA	0.2-0.001563	0.9978	115.7	0.2-0.001563	0.9949	149.3	3.2-0.0125	0.9961	13.7
TMAO	0.2-0.001563	0.9990	109.2	0.2-0.001563	0.9914	106.5	1.6-0.01250	0.9979	86.7
Norepinephrine	0.8-0.003125	0.9983	101.0	0.8-0.003125	0.9964	97.8	1.6-0.00625	0.9929	80.7
Epinephrine	0.4-0.00156	0.9984	104.0	0.4-0.00156	0.9974	130.5	1.6-0.001563	0.9943	99.3
L-valine	3.2-0.0125	0.9945	103.6	3.2-0.0125	0.9976	103.0	12.8-0.05	0.9974	103.3
Picolinic acid	0.4-0.006250	0.9983	99.4	0.4-0.006250	0.9955	97.4	0.8-0.00625	0.9932	92.3
Nicotinic acid	1.6-0.000781	0.9983	103.4	1.6-0.000781	0.9973	103.4	0.2-0.003125	0.9991	93.3
L-methionine-D_4	3.2-0.003125	0.9995	100.1	3.2-0.003125	0.9992	98.9	6.4-0.025	0.9998	101.5
L-methionine	3.2-0.001563	0.9987	106.2	3.2-0.001563	0.9998	104.7	3.4-0.0125	0.9997	100.8
Quinolinic acid	1.6-0.0125	0.9989	100.7	1.6-0.0125	0.9997	97.6	3.2-0.025	0.9957	97.5
L-Dopa	0.8-0.001563	0.9949	98.9	0.8-0.001563	0.9986	99.2	1.6-0.00625	0.9986	108.9
2-aminophenol	1.6-0.003125	0.9956	101.3	1.6-0.003125	0.9992	112.1	6.4-0.0125	0.9996	101.5
Dopamine- D_4	0.8-0.000781	0.9996	99.7	0.8-0.000781	0.9997	97.9	0.8-0.006250	0.9981	103.9
Dopamine	0.4-0.000391	0.9998	103.8	0.4-0.000391	0.9997	102.1	0.8-0.003125	0.9967	100.5
3-hydroxykynurenine	0.4-0.001563	0.9941	99.7	0.4-0.001563	0.9990	103.6	1.6-0.006250	0.9996	102.7
L-isoleucine-D_{10}	1.6-0.001563	0.9988	99.1	1.6-0.001563	0.9984	100.2	3.2-0.003125	0.9995	111.4
L-isoleucine	3.2-0.01250	0.9991	96.7	3.2-0.01250	0.9988	102.2	3.2-0.0125	0.9980	97.1

L-tyrosine-D_4	3.2-0.001560	0.9996	100.6	3.2-0.001560	0.9994	99.2	6.4-0.025	0.9978	97.6
L-tyrosine	6.4-0.006250	0.9985	101.8	6.4-0.006250	0.9985	98.2	6.4-0.00625	0.9981	86.8
Tyramine	0.8-0.000098	0.9997	98.7	0.8-0.000098	0.9993	98.0	0.4-0.003125	0.9979	81.5
L-leucine-D_{10}	3.2-0.001563	0.9990	101.6	3.2-0.001563	0.9980	99.4	3.2-0.001563	0.9996	99.8
L-leucine	3.2-0.01250	0.9972	98.8	3.2-0.01250	0.9977	101.1	3.2-0.0125	0.9911	90.0
Serotonin-D_4	0.8-0.001563	0.9978	93.2	0.8-0.001563	0.9921	90.2	0.4-0.006250	0.9920	100.0
Serotonin	0.2-0.001563	0.9987	106.2	0.2-0.001563	0.9984	112.1	0.4-0.001563	0.9910	98.6
3-methoxy-*p*-tyramine	0.1-0.000391	0.9967	95.8	0.1-0.000391	0.9960	98.1	0.2-0.001563	0.9990	106.4
5-hydroxy-L-tryptophan	0.8-0.003125	0.9961	100.6	0.8-0.003125	0.9988	99.8	3.2-0.01250	0.9994	97.1
N-methylserotonin	0.2-0.000781	0.9990	82.63	0.2-0.000781	0.99717	83.80	0.4-0.000781	0.9996	95.6
L-kynurenine-D_4	0.2-0.001563	0.9972	97.2	0.2-0.001563	0.9967	93.6	1.6-0.0125	0.9978	98.0
L-kynurenine	3.2-0.000781	0.9990	100.6	3.2-0.000781	0.9994	100.7	6.4-0.006250	0.9995	99.0
L-phenylalanine	6.4-0.0125	0.9990	98.0	6.4-0.0125	0.9988	95.3	3.2-0.00625	0.9964	92.2
4-hydroxyphenylacetylglycine	0.8-0.00156	0.9991	94.8	0.8-0.00156	0.9988	111.3	1.6-0.000781	0.9992	99.5
3-hydroxyanthranilic acid	0.8-0.003125	0.9961	97.1	0.8-0.003125	0.9972	112.7	3.2-0.025	0.9995	94.4
L-tryptophan-D_5	0.2-0.001563	0.9965	103.1	0.2-0.001563	0.9953	97.9	3.2-0.025	0.9993	96.5
Xanthurenic acid-D_4	0.1-0.000781	0.9938	112.3	0.1-0.000781	0.9962	109.9	3.2-0.003125	0.9997	104.2
L-tryptophan	3.2-0.003125	0.9960	100.3	3.2-0.003125	0.9980	100.1	3.2-0.006250	0.9954	87.8
Xanthurenic acid	0.1-0.000391	0.9952	90.9	0.1-0.000391	0.9946	90.7	6.4-0.0125	0.9999	101.2
3,4-dihydroxyphenylacetic acid-D_5	3.2-0.05	0.9963	98.8	3.2-0.05	0.9964	103.3	12.8-0.1	0.9983	97.6
3,4-dihydroxyphenylacetic acid	25.6-0.05	0.9969	96.0	25.6-0.05	0.9974	101.3	51.2-0.8	0.9988	104.4
3-(4-hydroxyphenyl)-lactic acid	3.2-0.006250	0.9997	99.3	3.2-0.006250	0.9990	99.6	6.4-0.05	0.9985	90.4
Homovanillic acid sulfate	3.2-0.003125	0.9994	101.4	3.2-0.003125	0.9994	106.5	3.2-0.006250	0.9991	101.9
N-acetyl-L-tyrosine	0.4-0.000391	0.9991	102.4	0.4-0.000391	0.9995	103.8	0.8-0.0125	0.9984	96.5
L-tryptophanol	0.2-0.000195	0.9956	106.5	0.2-0.000195	0.99451	105.9	0.4-0.000781	0.9975	96.7
Kynurenic acid-D_5	0.1-0.000391	0.9994	88.6	0.1-0.000391	0.9975	92.0	0.2-0.000781	0.9979	102.2
Kynurenic acid	0.4-0.000391	0.9981	109.1	0.4-0.000391	0.9996	113.3	0.8-0.000781	0.9977	90.5
5-methoxy-L-tryptophan	0.1-0.000391	0.9971	94.8	0.1-0.000391	0.9963	94.0	1.6-0.001563	0.9996	105.4
Tryptamine	0.8-0.000781	0.9983	95.8	0.8-0.000781	0.9977	98.90	0.2-0.000781	0.9993	84.8

4-hydroxyphenylpropionyl glycine	0.4-0.000781	0.9964	94.7	0.4-0.000781	0.9970	100.9	0.8-0.000781	0.9973	93.7
5-hydroxytryptophol	3.2-0.0125	0.9924	110.2	3.2-0.0125	0.99386	114.60	6.4-0.025	0.9996	97.4
5-methoxytryptamine	0.2-0.000098	0.9987	98.8	0.2-0.000098	0.9993	98.7	0.8-0.001563	0.9990	98.6
6-sulfathoxymelatonin	0.4-0.000195	0.9987	106.5	0.4-0.000195	0.9987	106.3	0.4-0.003125	0.9983	81.5
5-hydroxyindole-3-acetid acid-D_5	0.4-0.001563	0.9963	109.5	0.4-0.001563	0.9973	114.3	0.4-0.003125	0.9989	103.2
5-hydroxyindole-3-acetid acid	0.2-0.003125	0.9989	108.4	0.2-0.003125	0.9999	118.7	1.6-0.003125	0.9980	95.3
Indoxyl-β-glucoside	1.6-0.00625	0.9988	94.8	1.6-0.00625	0.9981	105.2	3.2-0.05	0.9995	92.6
N-acetyl-5-hydroxytryptamine	0.2-0.000195	0.9968	104.4	0.2-0.000195	0.9971	104.6	16-0.0125	0.9998	101.0
Indoxyl-β-glucuronide	1.6-0.00625	0.9990	97.4	1.6-0.00625	0.9985	101.6	1.6-0.0125	0.9947	93.6
Indoxyl sulfate-D_4	3.2-0.001560	0.9991	99.1	3.2-0.001560	0.9987	99.1	3.2-0.006250	0.9991	94.2
Indoxyl sulfate	1.6-0.001560	0.9993	104.1	1.6-0.001560	0.9996	103.4	1.6-0.001563	0.9999	86.9
Phenylacetyl-L-glutamine	3.2-0.000781	0.9997	98.5	3.2-0.000781	0.9996	94.1	3.2-0.00078	0.9921	93.8
Hippuric acid-D_5	1.6-0.000781	0.9990	99.8	1.6-0.000781	0.9972	97.6	3.2-0.00625	0.9992	97.9
Hippuric acid	3.2-0.003125	0.9966	101.4	3.2-0.003125	0.9979	107.7	25.6-0.025	0.9926	96.3
L-tryptophan, methyl ester	0.8-0.003125	0.9987	100.3	0.8-0.003125	0.9998	94.0	0.8-0.000781	0.9998	102.5
Homovanillic acid	3.2-0.025	0.9994	102.1	3.2-0.025	0.9987	103.0	12.8-0.2	0.9989	87.2
Phenylacetylglycine	0.8-0.000391	0.9992	96.2	0.8-0.000391	0.9996	105.8	0.4-0.000781	0.9955	85.6
Indole-3-acetyl aspartic acid	0.8-0.000195	0.9980	94.9	0.8-0.000195	0.9964	94.1	1.6-0.0125	0.9997	100.1
3-(4-hydroxyphenyl)-propionic acid	102.4-1.6	0.9989	82.7	102.4-1.6	0.99324	90.9	204.8-51.2	0.9999	91.2
p-cresol glucuronide	1.6-0.003125	0.9997	100.0	1.6-0.003125	0.9989	98.9	6.4-0.003125	0.9941	100.7
Indole-3-acetamide	0.4-0.00156	0.9959	100.4	0.4-0.00156	0.9955	97.9	0.8-0.001563	0.9992	103.5
L-tryptophan, ethyl ester	0.2-0.000098	0.9943	100.4	0.2-0.000098	0.9938	100.1	0.4-0.000391	0.9989	97.9
4-hydroxycinnamic acid	1.6-0.006250	0.9997	99.8	1.6-0.006250	0.9989	101.5	3.2-0.025	0.9996	96.7
p-cresol sulfate	3.2-0.001560	0.9984	101.3	3.2-0.001560	0.9982	99.8	12.8-0.003125	0.9956	93.1
Indole-3-acetylglutamic acid	0.4-0.000391	0.9996	100.9	0.4-0.000391	0.9987	104.7	1.6-0.001563	0.9996	103.2
Anthranilic acid	0.8-0.001563	0.9989	105.7	0.8-0.001563	0.9989	109.1	1.6-0.003125	0.9998	98.4
N-acetyl-L-phenylalanine	1.6-0.000781	0.9987	101.0	1.6-0.000781	0.9989	100.2	3.2-0.0125	0.9983	93.9
Phenyllactic acid	3.2-0.001560	0.9995	101.1	3.2-0.001560	0.9996	103.0	6.4-0.1	0.9995	91.3
N-acetyl-L-tryptophan	0.4-0.000391	0.9993	99.5	0.4-0.000391	0.9993	101.0	1.6-0.025	0.9992	95.8

Indole-3-lactic acid	3.2-0.000391	0.9995	101.2	3.2-0.000391	0.9993	102.0	3.2-0.0125	0.9997	99.2
N-acetyl-L-tyrosine, ethyl ester	0.8-0.000098	0.9994	100.7	0.8-0.000098	0.9997	98.7	1.6-0.001563	1.0000	103.2
Phenylpropionylglycine	0.2-0.000391	0.9967	97.6	0.2-0.000391	0.9964	98.4	1.6-0.000781	0.9985	93.8
Indole-3-acryloylglycine	0.4-0.001563	0.9996	113.4	0.4-0.001563	0.9994	119.6	1.6-0.000781	0.9960	91.7
Indole-3-carboxylic acid	0.4-0.003125	0.9974	117.0	0.4-0.003125	0.9985	109.6	3.2-0.1	0.9978	114.0
Cinnamoylglycine	1.6-0.000391	0.9990	100.3	1.6-0.000391	0.9991	97.5	3.2-0.003125	0.9997	98.4
Indole-3-carboxaldehyde	0.8-0.003125	0.9957	104.8	0.8-0.003125	0.9968	109.0	1.6-0.006250	0.9985	100.2
Melatonin	0.4-0.000781	0.9996	109.9	0.4-0.000781	0.9998	117.8	0.4-0.001563	0.9971	99.4
5-methoxytryptophol	0.4-0.000391	0.9983	100.9	0.4-0.000391	0.99543	87.85	3.2-0.0125	0.9994	97.7
5-methoxyindole-3-acetic acid	0.2-0.000391	0.9958	108.7	0.2-0.000391	0.99453	100.09	1.6-0.0125	0.9997	109.8
Benzoic acid	204.8-1.6	0.9993	87.0	204.8-1.6	0.99872	85.9	204.8-6.4	0.9991	93.6
Indole-3-acetic acid-D_5	3.2-0.000391	0.9996	104.8	3.2-0.000391	0.9994	103.0	6.4-0.006250	0.9997	97.1
Indole-3-acetic acid	1.6-0.000391	0.9973	100.7	1.6-0.000391	0.9971	99.7	6.4-0.001563	0.9988	96.2
Indole-3-ethanol	1.6-0.003125	0.9981	104.6	1.6-0.003125	0.9994	107.8	3.2-0.05	0.9995	96.1
Cinnabarinic acid	0.8-0.003125	0.9983	107.3	0.8-0.003125	0.9958	105.3	3.2-0.025	0.9999	96.6
Indole-3-acrylic acid	1.6-0.000391	0.9988	101.9	1.6-0.000391	0.9990	103.3	3.2-0.0125	0.9998	97.0
Indole-3-propionic acid	0.4-0.000391	0.9973	97.7	0.4-0.000391	0.9978	96.6	3.2-0.0125	0.9994	102.7
trans-cinnamic acid-D_5	0.4-0.000781	0.9973	98.1	0.4-0.000781	0.9971	96.6	6.4-0.1	0.9998	98.0
trans-cinnamic acid	3.2-0.00625	0.9998	101.6	3.2-0.00625	0.9971	103.6	6.4-0.4	0.9999	91.6
N-acetyl-L-tryptophan, ethyl ester	0.2-0.000098	0.9967	99.7	0.2-0.000098	0.9961	96.3	0.2-0.001563	0.9995	102.2
Indole-3-butyric acid	0.8-0.001563	0.9989	100.0	0.8-0.001563	0.9986	99.9	6.4-0.006250	0.9999	98.6
Indole-3-acetonitrile	0.4-0.000391	0.9996	102.1	0.4-0.000391	0.9996	106.7	1.6-0.0125	0.9995	99.7
Indole-3-acetic acid, methyl ester	0.05-0.000098	0.9979	99.0	0.05-0.000098	0.9971	103.6	0.8-0.001563	0.9976	100.4
Indole	12.8-0.4	0.9998	113.6	12.8-0.4	0.9995	140.1	102.4-0.8	0.9999	100.4
Indole-3-acetic acid, ethyl ester	0.1-0.000098	0.9970	98.9	0.1-0.000098	0.9960	100.6	1.6-0.000781	0.9995	97.6
Tryptanthrin	0.4-0.000098	0.9994	107.8	0.4-0.000098	0.9980	111.5	1.6-0.003125	0.9998	103.1
3-methylindole	12.8-0.2	0.9948	103.6	12.8-0.2	0.9997	107.8	51.2-0.4	0.9997	97.1

Supporting Table 5. Results of analytical recovery in plasma, serum and urine spiked at low, medium and high metabolite concentrations. For plasma and serum, analytical recoveries were calculated by calculating ratio of metabolite concentrations in pre-extraction spiking to concentrations in post-extraction spiked samples (*n*= 7). Post extraction spiking in urine was performed after urine filtration.

Name	PLASMA			SERUM			URINE		
	LOW	MED	HIGH	LOW	MED	HIGH	LOW	MED	HIGH
Histamine	60.9	72.4	72.8	68.7	81.9	88.2	85.4	93.4	93.3
Histidine	93.7	97.8	77.8	100.5	92.6	73.5	85.8	83.5	32.3
TMA	81.4	81.6	80.2	84.3	86.4	90.9	101.8	92.2	95.7
GABA	94.9	93.9	85.1	87.1	92.3	99.3	103.7	105.5	101.9
TMAO	86.8	85.3	80.7	80.7	89.4	93.8	101.2	88.3	91.2
Norepinephrine	83.4	89.0	87.1	96.9	92.8	90.1	70.8	86.5	66.1
Epinephrine	102.6	94.3	80.6	98.1	104.7	99.9	85.0	100.7	97.9
L-valine	96.2	104.0	96.9	108.1	100.5	95.1	104.0	100.4	111.7
Picolinic acid	81.6	96.7	83.3	80.9	95.0	98.4	100.4	96.9	86.4
Nicotinic acid	95.1	83.3	86.9	95.7	89.2	97.1	87.6	98.6	98.5
L-methionine-D_4	81.0	86.3	80.8	82.1	86.1	93.0	99.3	105.4	99.3
L-methionine	91.2	95.1	81.8	83.5	98.6	82.9	94.6	97.4	107.1
Quinolinic acid	80.1	77.3	75.7	73.1	76.8	81.3	82.1	99.5	96.2
L-Dopa	85.9	82.9	82.4	82.9	85.7	86.4	100.7	97.9	58.7
2-aminophenol	80.6	89.4	87.2	72.2	90.6	99.3	106.9	109.4	102.8
Dopamine- D_4	88.4	95.4	90.1	90.5	97.7	99.3	107.7	104.4	99.5
Dopamine	84.6	91.2	89.9	85.9	98.3	96.6	84.8	98.2	102.3
3-hydroxykynurenine	81.3	81.1	80.3	80.2	88.9	86.1	85.7	93.7	58.0
L-isoleucine-D_{10}	80.4	82.2	80.5	81.4	88.1	87.7	97.4	100.7	97.5

L-isoleucine	90.1	94.2	82.4	92.7	99.6	88.0	97.7	99.5	82.7
L-tyrosine-D_4	82.3	84.7	81.9	82.1	86.9	86.7	94.5	102.7	99.3
L-tyrosine	94.2	99.9	89.5	94.1	98.8	90.3	92.6	92.9	98.1
Tyramine	87.2	92.7	87.2	90.6	96.0	97.8	93.5	95.9	91.8
L-leucine-D_{10}	80.5	85.9	80.2	83.5	90.0	91.3	99.4	98.8	96.2
L-leucine	94.1	103.2	95.5	95.2	102.2	91.0	91.6	92.8	100.4
Serotonin-D_4	80.1	96.1	85.5	92.0	93.2	96.4	101.1	104.8	107.0
Serotonin	89.8	96.5	82.7	88.2	99.0	96.5	91.6	88.7	89.2
3-methoxy-*p*-tyramine	92.2	92.9	88.6	93.3	94.9	84.0	100.5	94.4	103.2
5-hydroxy-L-tryptophan	80.2	84.2	73.8	81.6	90.6	88.5	95.1	101.2	95.4
N-methylserotonin	85.0	86.5	87.4	83.4	103.9	93.9	99.6	98.6	98.5
L-kynurenine-D_4	71.5	81.5	81.0	82.1	85.0	90.7	104.0	90.1	90.5
L-kynurenine	80.0	92.3	78.1	83.3	92.0	83.0	98.4	83.9	114.1
L-phenylalanine	91.4	96.4	84.7	88.6	103.0	89.5	90.3	90.1	101.6
4-hydroxyphenylacetylglycine	93.7	111.5	92.5	91.5	102.5	106.9	107.8	100.9	103.4
3-hydroxyanthranilic acid	83.8	93.0	90.1	81.2	98.0	101.3	111.5	103.3	101.4
L-tryptophan-D_5	80.4	82.3	84.7	80.7	83.0	93.2	102.9	104.7	95.0
Xanthurenic acid-D_4	75.1	81.2	73.9	71.8	82.7	73.5	102.7	96.2	101.7
L-tryptophan	94.7	98.3	90.1	99.9	101.4	94.3	82.5	87.3	111.4
Xanthurenic acid	73.9	76.3	72.4	72.4	81.0	74.6	109.4	104.7	102.6
3,4-dihydroxyphenylacetic acid-D_5	92.3	84.8	103.1	99.9	86.5	87.2	102.7	99.8	100.6
3,4-dihydroxyphenylacetic acid	102.0	87.0	105.9	90.3	86.3	88.8	100.0	94.3	98.6
3-(4-hydroxyphenyl)-lactic acid	96.8	103.8	99.5	95.7	84.5	93.4	83.9	105.1	95.7
Homovanillic acid sulfate	97.8	100.3	99.3	93.4	99.8	95.9	102.0	88.5	96.8
N-acetyl-L-tyrosine	100.4	104.0	95.1	98.8	105.6	102.5	88.3	93.1	102.1
L-tryptophanol	93.1	90.5	88.1	93.9	92.6	95.3	100.8	94.7	101.3
Kynurenic acid-D_5	75.8	76.2	72.5	77.4	75.2	76.9	105.0	101.8	105.2
Kynurenic acid	73.4	74.9	66.1	66.9	85.1	68.7	90.3	108.6	95.3
5-methoxy-L-tryptophan	80.1	82.2	80.0	82.7	85.7	88.6	99.3	101.9	101.2
Tryptamine	92.8	92.9	91.9	94.9	99.9	96.9	89.7	86.2	102.3
4-hydroxyphenylpropionylglycine	104.2	102.4	94.2	102.0	101.6	103.9	102.1	99.6	104.7
5-hydroxytryptophol	96.7	98.0	96.9	94.0	97.8	100.2	116.9	99.5	92.3
5-methoxytryptamine	85.7	90.7	82.4	90.2	94.1	95.1	110.9	98.3	104.6

6-sulfathoxymelatonin	102.6	91.3	112.2	97.7	91.9	128.0	100.2	85.8	86.9
5-hydroxyindole-3-acetid acid-D_5	101.5	104.6	99.7	116.1	94.0	99.9	91.7	99.8	107.5
5-hydroxyindole-3-acetid acid	107.6	123.2	116.2	103.8	113.9	108.1	88.8	99.0	98.0
Indoxyl-*β*-glucoside	88.5	103.4	90.7	92.1	101.6	98.2	106.2	93.2	92.1
N-acetyl-5-hydroxytryptamine	96.0	101.0	94.2	91.0	99.6	99.9	116.5	101.4	97.0
Indoxyl-*β*-glucuronide	81.1	86.3	87.5	83.0	91.2	93.7	96.4	92.1	94.8
Indoxyl sulfate-D_4	104.9	92.3	103.6	98.4	89.5	91.9	89.6	86.6	90.9
Indoxyl sulfate	92.7	96.7	98.6	102.5	92.1	99.5	85.8	89.0	104.5
Phenylacetyl-L-glutamine	96.2	99.6	93.6	95.6	101.8	100.7	96.4	84.5	74.3
Hippuric acid-D_5	99.1	100.7	94.2	99.9	102.4	102.3	97.0	98.3	98.1
Hippuric acid	99.7	104.6	93.7	89.2	103.0	100.2	111.5	110.9	100.4
L-tryptophan, methyl ester	85.9	90.7	87.8	95.5	97.9	95.7	120.5	100.6	108.1
Homovanillic acid	98.2	104.3	102.4	102.8	102.1	89.6	95.4	94.6	98.0
Phenylacetylglycine	101.4	105.1	96.1	92.2	101.7	104.5	98.8	97.7	96.8
Indole-3-acetyl aspartic acid	91.3	94.6	90.2	94.9	98.8	100.7	92.7	99.6	102.7
3-(4-hydroxyphenyl)-propionic acid	77.6	98.3	86.3	79.1	90.2	84.9	106.2	106.9	101.6
p-cresol glucuronide	90.2	95.4	86.0	94.0	104.5	95.4	105.7	116.2	109.7
Indole-3-acetamide	92.6	103.9	95.1	86.7	97.3	95.3	89.0	102.2	109.0
L-tryptophan, ethyl ester	87.6	91.0	86.0	88.5	93.3	96.3	119.5	92.3	105.9
4-hydroxycinnamic acid	98.3	101.3	98.7	93.8	104.7	104.2	104.6	93.3	95.1
p-cresol sulfate	109.2	100.7	98.2	103.3	101.0	103.4	101.8	102.5	105.8
Indole-3-acetylglutamic acid	106.4	107.4	96.9	98.0	111.9	111.9	117.1	102.7	88.5
Anthranilic acid	80.5	95.8	88.6	88.5	100.0	102.6	86.1	92.6	105.1
N-acetyl-L-phenylalanine	99.1	103.1	94.0	98.9	106.0	97.7	110.3	83.6	90.8
Phenyllactic acid	108.7	100.0	100.0	102.8	95.3	97.6	101.6	97.3	98.4
N-acetyl-L-tryptophan	92.1	105.4	93.0	92.1	106.0	102.0	98.7	106.3	103.9
Indole-3-lactic acid	100.6	113.2	99.9	102.9	107.9	105.0	96.3	91.5	104.6
N-acetyl-L-tyrosine, ethyl ester	99.4	100.0	91.2	90.9	101.4	106.1	108.8	105.5	101.8
Phenylpropionylglycine	101.7	107.9	107.1	103.4	107.5	103.9	105.6	91.4	94.3
Indole-3-acryloylglycine	87.0	109.4	107.6	80.0	103.5	106.0	101.1	86.0	106.2
Indole-3-carboxylic acid	93.9	106.1	95.3	86.2	105.9	92.2	103.0	106.6	105.3
Cinnamoylglycine	97.6	104.2	93.8	97.0	104.9	100.6	107.2	93.8	94.6
Indole-3-carboxaldehyde	85.8	92.3	103.8	102.3	94.0	103.4	99.2	113.0	107.3

Melatonin	92.4	101.5	97.8	92.1	97.8	98.8	126.6	104.6	99.7
5-methoxytryptophol	96.2	92.9	87.5	95.5	90.2	102.2	96.1	99.1	102.8
5-methoxyindole-3-acetic acid	107.6	75.3	108.9	109.2	106.7	104.2	107.4	98.7	103.8
Benzoic acid	70.3	84.8	82.3	75.3	98.8	85.8	101.5	91..6	95.4
Indole-3-acetic acid-D_5	107.8	112.8	99.6	104.2	105.8	107.3	98.9	96.3	95.3
Indole-3-acetic acid	101.2	102.3	100.9	106.3	107.3	101.5	83.0	90.5	93.5
Indole-3-ethanol	96.2	91.8	81.7	98.5	93.0	102.9	110.9	100.4	94.3
Cinnabarinic acid	78.9	81.4	88.2	78.0	88.4	94.2	109.5	103.6	101.5
Indole-3-acrylic acid	80.4	98.5	92.8	73.4	99.6	103.4	97.6	105.0	97.5
Indole-3-propionic acid	97.8	108.5	98.2	100.2	110.6	108.3	98.8	96.3	107.0
trans-cinnamic acid-D_5	86.3	101.9	100.4	95.3	96.0	108.0	100.7	96.7	98.6
trans-cinnamic acid	86.7	82.4	90.9	97.0	93.3	102.1	97.9	99.2	106.2
N-acetyl-L-tryptophan, ethyl ester	93.9	94.0	78.2	97.2	96.6	104.5	93.6	91.6	100.1
Indole-3-butyric acid	94.5	110.2	100.5	93.4	110.9	105.7	104.6	97.4	100.5
Indole-3-acetonitrile	104.1	107.9	96.6	105.2	84.4	100.1	106.8	91.4	99.6
Indole-3-acetic acid, methyl ester	80.7	81.0	81.7	83.2	77.1	83.2	106.4	96.3	110.2
Indole	25.6	30.9	40.6	49.5	40.3	67.1	102.0	105.0	107.3
Indole-3-acetic acid, ethyl ester	88.1	96.6	95.1	97.0	79.6	93.0	102.0	90.1	97.0
Tryptanthrin	87.9	91.0	88.0	89.6	78.4	103.1	115.2	93.3	103.0
3-methylindole	30.2	29.8	58.8	69.1	35.7	66.7	101.7	112.0	104.0

Supporting Table 6. Results of analytical accuracy in plasma, serum and urine spiked at medium concentrations (n = 7) at day-1, 3 and 5 and inter-day variability (I.D.). Accuracy is calculated as (detected concentration/spiked concentration) * 100 ; CV% are reported in brackets.

Name	PLASMA				SERUM				URINE			
	DAY 1	DAY 3	DAY 5	I.D.	DAY 1	DAY 3	DAY 5	I.D.	DAY 1	DAY 3	DAY 5	I.D.
Histamine	0.6 (2.8)	2.2 (3.9)	-0.6 (1.9)	3.1	4.9 (1.3)	3.7 (0.9)	-2.6 (3.0)	3.0	4.2 (2.2)	3.5 (3.6)	4.2 (3.6)	3.0
Histidine	-1.1 (6.1)	0.6 (4.8)	-0.5 (4.0)	4.9	0.2 (6.5)	-1.1 (6.6)	4.0 (13.3)	7.6	-0.4 (1.5)	0.5 (1.1)	0.4 (1.1)	1.3
TMA	2.8 (6.1)	-1.7 (6.9)	-0.1 (1.9)	5.7	-2.8 (2.8)	1.3 (4.2)	0.0 (0.9)	3.7	2.2 (8.1)	1.0 (3.9)	-4.5 (4.1)	6.3
GABA	-3.0 (8.9)	-0.5 (7.8)	3.8 (5.6)	3.6	0.8 (7.4)	1.6 (5.6)	-0.3 (2.7)	4.7	1.1 (7.1)	-6.9 (10.7)	-9.8 (11.0)	10.5
TMAO	-3.9 (2.0)	1.6 (3.0)	5.0 (2.1)	8.1	-3.0 (4.3)	0.5 (5.2)	4.6 (5.7)	6.4	7.3 (15.7)	9.1 (5.9)	6.8 (6.0)	9.6
Norepinephrine	-1.3 (2.9)	0.6 (3.9)	-5.3 (2.1)	3.9	-0.5 (5.8)	1.2 (2.8)	3.2 (7.6)	5.0	5.3 (15.5)	-3.5 (17.8)	-1.4 (17.4)	20.3
Epinephrine	-6.8 (7.9)	-5.4 (9.9)	0.9 (3.5)	8.1	2.4 (3.6)	-7.9 (3.8)	-7.1 (2.9)	6.3	4.9 (8.1)	-0.2 (11.0)	-5.5 (11.6)	9.2
L-valine	0.7 (8.8)	3.2 (9.2)	8.4 (7.6)	8.6	3.4 (5.3)	-1.6 (5.7)	-3.5 (14.0)	3.7	3.8 (12.0)	-4.5 (13.7)	-8.7 (14.3)	12.7
Picolinic acid	-3.1 (5.0)	-1.8 (2.6)	-2.8 (2.9)	3.6	-3.1 (5.0)	1.0 (3.3)	0.2 (3.3)	6.4	-3.7 (6.2)	-2.9 (3.3)	-2.0 (3.3)	4.5

Nicotinic acid	-1.2 (1.8)	4.3 (3.2)	4.6 (1.1)	3.5	2.4 (2.0)	7.5 (2.2)	7.1 (0.7)	3.0	0.6 (4.4)	-5.3 (6.1)	-4.3 (6.0)	6.5
L-methionine-D_4	-0.8 (2.1)	-1.1 (2.0)	-3.6 (2.3)	2.3	-2.4 (1.1)	1.5 (1.1)	-2.5 (1.3)	2.3	2.8 (2.3)	3.5 (4.1)	2.7 (4.1)	3.4
L-methionine	-8.9 (11.2)	-3.3 (7.5)	-2.8 (8.3)	9.1	0.6 (2.6)	-0.6 (2.2)	-1.6 (0.9)	2.3	-1.1 (7.3)	-6.6 (6.4)	-5.6 (6.3)	10.4
Quinolinic acid	-4.5 (7.3)	-2.8 (3.0)	-8.1 (7.4)	6.1	-5.8 (2.3)	2.2 (3.8)	-2.7 (0.8)	4.7	-6.1 (3.2)	-1.8 (1.9)	-0.7 (1.9)	4.0
L-Dopa	-2.9 (3.9)	1.9 (7.6)	-5.5 (3.4)	6.2	-6.9 (4.0)	-0.4 (3.9)	-8.6 (1.7)	5.2	2.7 (3.5)	1.2 (2.4)	0.9 (2.4)	3.5
2-aminophenol	-2.9 (2.7)	-2.8 (8.9)	-0.1 (2.2)	5.6	-6.7 (5.9)	0.2 (3.3)	-3.3 (1.2)	5.2	-9.0 (8.2)	-10.7 (4.1)	-2.3 (7.4)	7.7
Dopamine- D_4	-3.0 (3.5)	-1.8 (5.7)	-5.3 (4.8)	4.6	-9.2 (4.6)	-4.5 (4.3)	-11.0 (2.0)	5.0	4.6 (4.0)	2.0 (3.6)	3.6 (3.5)	3.6
Dopamine	-3.5 (1.1)	-0.5 (2.7)	-3.1 (2.0)	2.4	-0.6 (2.5)	-4.5 (3.3)	-5.8 (2.5)	3.5	1.2 (4.8)	4.3 (2.2)	6.0 (2.1)	6.8
3-hydroxykynurenine	0.1 (9.4)	9.4 (4.7)	2.4 (9.0)	8.3	-5.6 (8.4)	-4.7 (8.6)	2.2 (2.8)	7.9	-6.8 (4.3)	-2.1 (3.2)	-1.5 (3.2)	4.1
L-isoleucine-D_{10}	-1.2 (1.8)	0.0 (2.7)	-2.9 (3.1)	2.7	-0.2 (1.5)	-0.1 (2.1)	-3.3 (1.1)	2.1	1.5 (3.6)	2.6 (3.9)	0.9 (3.9)	3.3
L-isoleucine	-2.6 (8.4)	-5.4 (6.4)	-3.5 (8.4)	7.4	-0.5 (7.9	-0.1 (2.3)	2.7 (12.2)	2.1	-3.2 (12.2)	-1.6 (10.7)	-3.1 (10.9)	11.6
L-tyrosine-D_4	-1.8 (1.7)	-2.1 (1.8)	-0.8 (2.8)	2.0	0.9 (1.8)	-4.5 (2.1)	-2.1 (0.3)	3.1	-1.7 (1.1)	2.7 (2.0)	2.2 (2.0)	2.5
L-tyrosine	-1.0 (6.1)	-3.0 (8.6)	-2.8 (2.3)	6.3	-4.4 (4.4)	-6.5 (3.8)	2.3 (0.5)	3.7	7.1 (5.6)	5.3 (4.9)	3.3 (5.0)	4.8
Tyramine	-3.0 (2.2)	4.1 (3.0)	-3.2 (2.2)	4.3	-2.2 (2.0)	3.2 (1.8)	-2.5 (0.7)	3.3	1.1 (11.2)	0.0 (6.4)	-1.4 (6.5)	8.2
L-leucine-D_{10}	-4.2 (2.0)	-0.9 (2.5)	-2.7 (1.7)	2.5	1.3 (1.2)	-0.1 (1.3)	-1.1 (1.4)	1.5	-4.6 (2.5)	-3.9 (1.7)	-6.7 (1.7)	4.5
L-leucine	-1.6 (5.0)	1.9 (11.9)	2.1 (8.9)	8.9	-2.6 (4.6)	-0.1 (4.9)	-4.7 (6.0)	6.0	-5.3 (4.1)	-1.4 (1.1)	-2.5 (1.1)	3.2
Serotonin-D_4	-4.6 (3.2)	-2.2 (6.7)	-5.0 (3.0)	4.8	-3.4 (7.6)	-1.8 (6.0)	1.1 (5.7)	6.5	-6.5 (3.9)	-4.0 (5.1)	-6.1 (5.2)	4.9
Serotonin	-6.3 (4.4)	-4.7 (7.8)	-4.0 (2.6)	5.4	-4.1 (3.7)	-1.2 (3.5)	-3.8 (2.1)	3.5	-5.0 (6.5)	-4.9 (7.7)	-9.3 (8.0)	8.8
3-methoxy-*p*-tyramine	0.6 (3.4)	-0.5 (3.7)	-5.4 (3.4)	4.2	3.8 (2.9)	-0.3 (1.9)	-1.5 (3.0)	3.3	-0.3 (3.2)	-5.4 (7.2)	-6.1 (7.2)	3.9
5-hydroxy-L-tryptophan	-11.6 (9.7)	-0.7 (5.2)	1.5 (2.2)	8.2	0.4 (2.7)	1.2 (4.8)	1.1 (0.8)	3.4	-5.5 (2.9)	-3.4 (8.5)	-9.2 (9.0)	7.3
N-methylserotonin	-6.2 (9.7)	-2.0 (7.8)	-0.2 (3.3)	7.7	-0.1 (3.7)	1.8 (4.5)	1.0 (3.7)	3.9	-2.6 (2.9)	-5.4 (3.7)	-8.2 (3.8)	3.9
L-kynurenine-D_4	2.2 (3.2)	2.5 (3.7)	-0.2 (3.9)	3.5	-1.9 (3.1)	-0.4 (4.4)	1.23 (2.3)	3.6	0.5 (5.9)	4.9 (3.6)	-0.6 (3.7)	5.8

L-kynurenine	-6.1 (4.2)	0.5 (5.3)	-1.2 (2.5)	5.1	-4.1 (2.3)	2.4 (3.3)	-5.0 (0.7)	4.3	-9.3 (5.2)	-8.7 (4.6)	-9.2 (4.6)	5.0
L-phenylalanine	-0.2 (4.6)	-2.5 (6.0)	-3.3 (5.6)	5.3	-5.2 (2.6)	2.1 (4.2)	2.6 (1.3)	4.9	-4.1 (6.0)	3.2 (4.1)	-1.2 (4.3)	5.3
4-hydroxyphenyl-acetylglycine	-5.2 (7.7)	-2.6 (11.9)	2.8 (5.5)	9.1	0.4 (6.2)	1.7 (4.5)	-3.0 (4.4)	5.2	-6.8 (2.1)	1.8 (7.4)	-1.7 (7.6)	5.7
3-hydroxy-anthranilic acid	-4.0 (3.5)	1.9 (4.7)	-1.6 (2.9)	4.5	-5.3 (3.4)	2.9 (3.1)	2.0 (0.8)	5.0	-0.2 (3.3)	-7.4 (12.8)	-8.4 (12.9)	11.1
L-tryptophan-D_5	-1.7 (1.8)	-3.3 (3.9)	0.0 (1.8)	2.9	-3.6 (2.5)	-3.7 (2.4)	-0.6 (1.7)	2.5	2.0 (3.5)	4.4 (2.4)	6.1 (2.4)	3.7
Xanthurenic acid-D_4	-10.8 (7.7)	-4.6 (4.0)	-1.9 (0.8)	6.4	-6.8 (3.2)	-1.0 (2.1)	-1.9 (0.6)		-3.0 (7.1)	-6.7 (3.9)	-7.1 (3.9)	6.0
L-tryptophan	-2.6 (4.9)	-3.1 (7.0)	3.5 (3.8)	5.9	0.8 (5.0)	-7.5 (6.5)	-5.8 (6.1)	6.9	8.0 (4.9)	-6.4 (4.2)	1.1 (3.9)	7.3
Xanthurenic acid	-2.2 (3.8)	-2.4 (4.8)	-5.7 (5.9)	4.8	-8.4 (2.1)	-3.5 (3.0)	-2.2 (0.6)	3.7	-1.5 (6.8)	-0.5 (4.2)	-4.6 (4.4)	5.4
3,4-dihydroxyphenyl acetic acid-D_5	-9.8 (3.7)	-5.8 (9.9)	-3.4 (6.5)	7.5	-3.9 (4.4)	-5.3 84.2)	-2.5 (0.6)	3.9	5.8 (4.4)	-3.0 (2.2)	-3.2 (2.2)	5.5
3,4-dihydroxyphenyl acetic acid	2.3 (8.6)	6.7 (6.9)	-2.8 (10.2)	8.7	-0.4 (6.9)	-0.9 (10.1)	-.2.4 (7.7)	8.0	2.6 (3.5)	0.9 (5.3)	-0.4 (5.3)	4.1
3-(4-hydroxyphenyl)-lactic acid	-3.7 (4.0)	-2.0 (5.1)	1.5 (13.4)	7.8	-1.2 (3.7)	0.7 (3.5)	2.7 (7.0)	4.3	-1.2 (4.9)	-4.4 (3.6)	-3.2 /3.5)	4.3
Homovanillic acid sulfate	0.4 (4.8)	-2.2 (8.8)	2.4 (5.0)	6.1	0.7 (5.6)	2.9 (5.5)	6.2 (0.8)	5.2	7.2 (2.1)	1.8 (5.6)	-0.6 (5.7)	4.9
N-acetyl-L-tyrosine	-3.5 (4.8)	0.3 (2.0)	0.8 (4.2)	4.1	-4.6 (5.0)	-0.1 (5.4)	5.0 (2.7)	5.8	3.0 (3.4)	-0.4 (4.4)	-4.0 (4.5)	4.6
L-tryptophanol	-2.6 (4.4)	-5.3 (2.0)	-2.4 (2.5)	3.3	0.4 (2.5)	-1.6 (2.4)	1.9 (4.9)	3.1	-2.1 (8.8)	-3.8 (3.1)	3.7 (2.9)	6.7
Kynurenic acid-D_5	1.9 (1.9)	2.4 (4.6)	-4.9 (1.9)	4.4	0.9 (1.6)	-1.4 (3.0)	-0.2 (2.9)	2.5	-4.0 (3.1)	-3.7 (1.0)	-3.4 (1.0)	1.9
Kynurenic acid	-8.6 (5.4)	-2.1 (7.1)	2.3 (3.7)	7.1	-3.3 (7.5)	-1.0 (4.4)	0.4 (3.7)	5.6	-5.6 (2.8)	-2.2 (1.4)	-0.8 (1.3)	2.9
5-methoxy-L-tryptophan	-0.9 (2.9)	-0.2 (2.0)	0-2.0 (1.9)	2.3	-4.1 (3.1)	-2.8 (3.1)	-5.4 (6.5)	3.7	-0.8 (5.7)	3.5 (1.5)	2.6 (1.5)	4.7
Tryptamine	-6.6 (2.5)	-3.9 (4.7)	-1.5 (2.7)	4.0	-2.6 (5.1)	-5.7 (4.8	-0.5 (2.7)	4.8	-4.3 (3.0)	-1.1 (1.3)	-3.4 (1.3)	2.5
4-hydroxyphenyl-propionylglycine	3.6 (3.4)	-1.0 (3.8)	2.2 (2.4)	3.7	1.5 (3.5)	5.0 (0.9)	3.7 (6.4)	3.5	-4.5 (6.6)	2.8 (3.5)	1.4 (3.5)	5.1
5-hydroxytryptophol	0.4 (2.0)	-1.7 (3.6)	-0.2 (4.8)	3.4	-2.1 (1.3)	-0.9 (2.8)	-1.4 (5.0)	2.7	2.1 (2.3)	0.1 (1.2)	3.5 (1.1)	2.6
5-methoxytryptamine	-3.8 (2.1)	-3.4 (4.9)	-6.5 (1.8)	3.5	-5.0 (1.6)	-2.2 (3.0)	-6.3 (2.6)	2.9	2.0 (6.2)	4.8 (3.2)	5.0 (3.2)	4.6
6-sulfathoxy-melatonin	-6.5 (6.7)	-0.8 (11.7)	-2.9 (8.9)	9.3	-5.3 (4.1)	-11.5 (5.0)	3.2 (0.7)	7.7	-7.0 (3.1)	-2.9 (4.0)	-4.2 (4.1)	3.8

5-hydroxyindole-3-acetid acid-D_5	-2.4 (4.4)	-2.4 (3.9)	2.2 (2.4)	4.1	-1.8 (.4)	-2.6 (3.3)	0.3 (3.0)	3.7	-1.8 (2.1)	-4.2 (4.0)	-2.0 (4.0)	3.8
5-hydroxyindole-3-acetid acid	-7.6 (6.1)	0.1 (5.5)	-2.5 (4.7)	6.5	-6.9 (4.6)	1.2 (3.9)	-0.6 (3.0)	5.5	4.1 (9.1)	1.5 (7.2)	-4.1 (7.6)	8.8
Indoxyl-*β*-glucoside	-8.7 (5.8)	1.5 (4.6)	-3.0 (8.9)	7.6	-5.1 (8.4)	1.5 (4.6)	3.7 (7.3)	7.4	-1.7 (2.9)	-3.6 (5.2)	-2.1 (5.1)	5.4
N-acetyl-5-hydroxy-tryptamine	-3.0 (2.1)	-4.4 (1.8)	-4.0 (2.6)	2.1	-5.0 (2.2)	-3.1 (1.3)	-2.9 (1.2)	2.0	0.8 (2.7)	1.4 (4.2)	-1.5 (4.4)	3.4
Indoxyl-*β*-glucuronide	-0.1 (6.6)	-4.4 (9.3)	-9.0 (3.5)	7.8	-0.8 (2.7)	-6.3 (8.5)	-9.4 (5.2)	6.7	0.4 (4.7)	-2.8 (2.8)	-5.4 (2.9)	3.8
Indoxyl sulfate-D_4	-4.4 (2.9)	-1.3 (2.2)	-2.9 (1.7)	2.6	-2.7 (1.9)	-4.0 (4.2)	-1.9 (0.4)	2.9	-5.1 (3.1)	-1.3 (3.2)	-2.2 (3.2)	3.2
Indoxyl sulfate	-7.0 (7.3)	-2.3 (1.6)	-2.7 (3.5)	5.1	-3.9 (2.1)	-6.5 (4.8)	-0.6 (2.2)	4.0	0.1 (4.3)	-0.6 (1.8)	-1.7 (1.8)	3.1
Phenylacetyl-L-glutamine	-1.6 (1.3)	-1.7 (5.1)	-6.6 (5.4)	4.6	-6.0 (4.0)	-7.2 (2.8)	-3.9 (4.9)	3.7	2.1 (1.6)	-1.1 (1.8)	0.2 (1.8)	2.2
Hippuric acid-D_5	0.4 (2.2)	1.2 (1.7)	-3.9 (4.4)	3.4	0.6 (0.7)	-2.7 (2.1)	-2.2 (0.9)	2.2	-0.9 (2.1)	4.0 (1.6)	2.1 (1.6)	2.6
Hippuric acid	-3.2 (2.9)	-1.2 (3.6)	-2.3 (2.2)	3.0	-4.5 (6.7)	0.8 (2.1)	0.1 (1.1)	5.0	-0.9 (2.2)	0.5 (0.7)	0.2 (0.7)	1.4
L-tryptophan, methyl ester	-0.3 (2.9)	-6.2 (3.7)	-2.3 (3.4)	4.1	-4.1 (2.3)	-6.0 (2.9)	-3.4 (1.9)	2.6	-3.7 (3.8)	1.7 (4.1)	6.0 (3.9)	4.7
Homovanillic acid	-10.2 (6.8)	-7.2 (8.5)	-0.7 (5.7)	7.9	-5.2 (6.1)	-4.2 (11.1)	4.8 (4.4)	8.8	1.9 (2.5)	2.7 (2.5)	3.1 (2.5)	2.2
Phenylacetylglycine	-1.5 (4.9)	-1.1 (2.3)	2.1 (1.4)	3.5	1.4 (2.4)	3.3 (3.1)	6.5 (0.8)	3.0	0.3 (3.1)	-5.9 (3.6)	-4.7 (3.5)	5.3
Indole-3-acetyl-aspartic acid	-3.8 (3.0)	-1.7 (1.8)	-2.4 (1.7)	2.4	-1.1 (3.9)	-0.1 (1.2)	-6.0 (6.7)	4.0	-5.2 (1.7)	2.8 (3.6)	1.8 (3.7)	4.8
3-(4-hydroxyphenyl)-propionic acid	0.3 (4.9)	4.0 (2.3)	1.6 (3.6)	4.0	-10.0 (10.0)	-23.3 (56.9)	-1.9 (4.3)	29.8	-4.3 (6.3)	-1.9 (2.5)	-2.9 (2.5)	4.8
p-cresol glucuronide	-9.1 (4.2)	-2.1 (1.7)	-5.4 (7.6)	5.5	-4.2 (2.9)	-2.1 (1.7)	-4.9 (6.2)	3.2	-0.7 (1.4)	-0.6 (5.4)	0.6 (5.4)	4.3
Indole-3-acetamide	-0.7 (6.5)	-4.0 (10.2)	1.6 (3.0)	7.4	0.5 (3.6)	-2.0 (2.2)	-2.4 (1.8)	3.0	-2.2 (1.5)	-0.1 (3.2)	0.8 (3.2)	2.7
L-tryptophan, ethyl ester	0.3 (1.7)	2.0 (2.3)	-2.5 (1.6)	2.5	0.4 (1.1)	-1.3 (2.3)	-5.2 (2.2)	2.7	-4.9 (3.7)	6.9 (1.9)	4.5 (1.9)	5.5
4-hydroxycinnamic acid	-3.1 (7.9)	-0.1 (6.4)	-1.0 (6.4)	6.8	-10.1 (6.0)	-4.5 (7.8)	-0.9 (4.8)	7.4	2.8 (2.5)	-3.9 (4.7)	1.3 (4.5)	5.0
p-cresol sulfate	-3.1 (5.3)	2.1 (9.4)	6.1 (9.1)	8.2	2.7 (7.3)	-6.0 (7.3)	-2.7 (9.0)	8.2	-2.1 (3.6)	5.4 (5.2)	3.6 (5.9)	7.0
Indole-3-acetyl-glutamic acid	-13.9 (3.2)	-10.5 (4.3)	-8.2 (3.7)	4.0	-13.5 (2.6)	-7.3 (1.7)	3.7 (0.8)	4.8	5.7 (8.9)	-3.0 (9.6)	1.4 (9.2)	10.8
Anthranilic acid	-11.3 (7.4)	-9.7 (7.6)	-0.4 (6.1)	8.4	-11.1 (4.5)	-0.5 (3.9)	-2.5 (7.0)	7.0	7.2 (3.5)	1.6 (2.5)	-1.9 (2.6)	4.9

N-acetyl-L-phenylalanine	-5.8 (2.7)	-1.4 (2.7)	-2.5 (5.3)	3.9	-6.3 (1.9)	-3.3 (2.3)	-2.9 (2.5)	2.6	9.0 (5.1)	8.6 (4.0)	7.0 (4.1)	4.0
Phenyllactic acid	-4.4 (3.0)	-0.7 (2.4)	-2.4 (6.8)	4.2	-3.8 (2.6)	-1.3 (2.2)	1.1 (1.0)	2.8	-1.2 (4.5)	-0.7 (2.6)	-4.6 (2.7)	4.3
N-acetyl-L-tryptophan	-3.9 (5.3)	2.3 (6.0)	2.3 (6.0)	6.2	-4.1 (6.5)	-4.5 (6.4)	2.9 (2.3)	6.3	-6.2 (7.0)	-4.7 (7.3)	-9.5 (7.7)	6.7
Indole-3-lactic acid	0.9 (1.2)	0.7 (1.4)	-1.3 (2.1)	1.8	2.4 (2.5)	-0.2 (2.4)	-0.4 (0.4)	2.5	2.7 (2.2)	-5.7 (6.6)	-5.9 (6.6)	5.9
N-acetyl-L-tyrosine, ethyl ester	-6.8 (3.7)	-4.2 (2.8)	-3.7 (2.6)	3.3	-6.6 (1.4)	-2.5 (2.0)	0.1 (3.4)	3.4	-6.9 (2.3)	5.0 (2.5)	4.3 (2.5)	5.9
Phenylpropionyl-glycine	-0.1 (2.0)	0.3 (3.1)	-0.6 (2.8)	2.5	-0.4 (0.8)	-0.4 (7.4)	1.0 (1.6)	4.6	-6.0 (2.9)	-1.9 (4.8)	-3.2 (4.8)	3.6
Indole-3-acryloylglycine	-10.0 (6.5)	-6.4 (12.5)	0.7 (3.2)	9.4	-9.1 (3.6)	5.2 (4.4)	5.1 (1.1)	8.1	-2.0 (10.0)	-2.2 (7.7)	-4.4 7.9)	8.8
Indole-3-carboxylic acid	-3.8 (6.5)	2.0 (7.6)	-0.4 (9.1)	7.6	2.6 (4.4)	-1.0 (3.4)	3.3 (1.6)	4.0	2.1 (2.9)	-5.1 (3.9)	-6.0 (3.0)	4.8
Cinnamoylglycine	-9.0 (1.5)	-7.4 (3.5)	-7.6 (1.5)	2.5	-11.3 (1.3)	-5.9 (1.7)	-5.1 (2.6)	3.5	-1.9 (5.5)	-2.0 (6.3)	-7.0 (6.6)	6.8
Indole-3-carboxaldehyde	-9.7 (4.9)	-7.9 (3.7)	-3.7 (7.9)	5.8	-3.0 (7.9)	6.6 (7.3)	5.3 (3.1)	8.0	3.1 (5.4)	-1.9 (4.8)	-2.0 (4.8)	6.4
Melatonin	0.1 (1.4)	-4.1 (1.0)	-6.4 (1.8)	3.1	-3.6 (1.5)	-3.6 (2.8)	0.7 (1.5)	2.6	-8.8 (3.9)	4.8 (2.6)	2.2 (2.7)	9.1
5-methoxytryptophol	2.9 (2.1)	1.8 (1.2)	-1.8 (1.4)	2.5	-3.1 (1.8)	-3.1 (1.5)	-1.3 (0.9)	1.6	-11.6 (3.1)	4.5 (3.2)	1.4 (3.3)	8.3
5-methoxyindole-3-acetic acid	0.5 (2.9)	0.1 (3.4)	3.3 (1.3)	3.0	-2.5 (2.1)	4.1 (1.4)	1.5 (3.2)	3.6	-3.7 (3.0)	2.9 (1.1)	1.0 (1.2)	3.8
Benzoic acid	-9.1 (18.7)	-6.0 (5.9)	-6.2 (9.0)	11.5	-15.7 (11.9)	-4.3 (15.1)	-1.6 (5.5)	13.7	2.1 (1.6)	1.2 (1.9)	0.4 (1.9)	2.1
Indole-3-acetic acid-D_5	1.0 (2.6)	4.9 (2.4)	3.3 (2.6)	2.9	-8.1 (3.0)	2.3 (1.6)	-1.8 (2.3)	5.5	0.8 (8.8)	1.7 (1.5)	2.3 (1.5)	2.2
Indole-3-acetic acid	0.0 (3.6)	1.8 (1.1)	-0.1 (3.5)	2.8	-0.7 (1.5)	-4.3 (2.3)	4.0 (2.1)	3.6	-4.7 (2.9)	-0.6 (1.7)	-0.2 (1.7)	3.0
Indole-3-ethanol	-0.5 (1.5)	-1.9 (1.6)	-1.1 (2.8)	2.0	-1.5 (0.8)	-0.6 (1.3)	-1.0 (0.6)	1.0	-2.9 (4.7)	-3.1 (5.3)	1.9 (5.1)	4.7
Cinnabarinic acid	-4.7 (3.2)	-2.1 (2.4)	-0.7 (1.3)	2.9	-1.6 (2.7)	0.0 (2.3)	0.1 (2.2)	2.4	-6.1 (12.1)	-8.4 (10.6)	-4.3 (9.7)	11.7
Indole-3-acrylic acid	-2.8 (1.4)	-5.5 (1.4)	4.3 (0.7)	4.3	-10.9 (1.7)	-10.6 (3.4)	-1.3 (5.0)	5.1	-3.0 (4.1)	4.8 (2.7)	1.7 (2.7)	4.2
Indole-3-propionic acid	-1.0 (1.4)	0.8 (2.1)	-2.7 (3.2)	2.5	1.1 (1.8)	-0.2 (1.1)	0.2 (2.8)	1.8	3.4 (3.9)	1.0 (3.4)	6.0 (3.3)	4.0
trans-cinnamic acid-D_5	-2.5 (2.9)	-1.1 (3.1)	1.3 (3.7)	3.4	-5.0 (2.1)	-2.8 (4.4)	1.1 (3.5)	4.0	-7.4 (3.8)	0.6 (2.3)	2.7 (2.3)	5.2
trans-cinnamic acid	-2.7 (2.1)	0.5 (3.7)	-0.8 (2.2)	3.0	-1.9 (3.3)	-2.7 (2.0)	4.1 (3.4)	3.7	2.1 (3.1)	-2.7 (4.1)	-8.0 (4.3)	6.1

N-acetyl-L-tryptophan, ethyl ester	-0.5 (5.9)	1.0 (4.2)	-1.7 (2.0)	4.4	-5.8 (2.4)	-6.9 (2.1)	-5.1 (0.9)	2.1	0.3 (3.4)	3.3 (2.8)	0.9 (2.9)	3.3
Indole-3-butyric acid	-2.2 (1.3)	-3.3 (3.2)	-7.5 (1.8)	3.2	-6.3 (1.3)	-0.7 (2.5)	-6.1 (1.3)	3.5	-1.9 (2.7)	-0.1 (1.1)	0.7 (1.1)	2.0
Indole-3-acetonitrile	-4.9 (2.5)	0.2 (2.8)	-3.9 (2.9)	3.5	-3.8 (1.8)	0.5 (1.1)	-4.2 (0.3)	2.6	0.3 (8.8)	0.3 (5.2)	-6.2 (5.5)	8.6
Indole-3-acetic acid, methyl ester	0.3 (3.3)	0.6 (3.7)	5.5 (1.7)	3.7	-0.4 (1.7)	1.4 (2.1)	3.9 (2.2)	2.4	6.2 (2.7)	1.6 (1.5)	4.1 (1.5)	2.7
Indole	-5.1 (2.9)	-3.7 (4.1)	4.2 (3.3)	5.2	1.0 (2.8)	2.2 (3.2)	2.4 (2.5)	2.8	2.5 (4.2)	-6.1 (2.5)	-3.2 (2.4)	6.1
Indole-3-acetic acid, ethyl ester	-7.1 (1.2)	-9.0 (3.5)	-5.9 (2.4)	2.8	-2.8 (1.5)	1.2 (1.5)	-3.1 (3.7)	2.8	-4.3 (3.0)	2.7 (2.1)	1.9 (2.1)	2.6
Tryptanthrin	1.6 (3.5)	-0.3 (1.3)	-1.4 (2.0)	2.7	1.2 (2.6)	-3.1 (1.1)	-1.5 (2.0)	2.8	2.5 (2.1)	-0.1 (5.0)	1.4 (4.9)	4.2
3-methylindole	-3.3 (1.3)	-0.5 (2.1)	1.8 (1.2)	2.6	-0.2 (0.9)	4.8 (3.6)	2.3 (2.3)	3.3	-9.4 (1.9)	-9.9 (3.2)	-14.7 (3.4)	3.9

Supporting Table 7. Intra-day precision, determined in three different days (1, 3 and 5), and inter-day (I.D.) accuracy, in plasma, serum and urine, expressed as CV%.

Name	PLASMA CV%				SERUM CV%				URINE CV%			
	DAY 1	DAY 3	DAY 5	I.D.	DAY 1	DAY 3	DAY 5	I.D.	DAY 1	DAY 3	DAY 5	I.D.
HSM	1.8	2.5	1.9	6.7	1.3	0.9	3.0	6.5	1.8	2.4	2.0	2.1
HSD	8.6	8.4	3.8	8.6	2.7	5.0	1.9	3.6	0.8	1.1	2.9	2.0
TMA	2.7	10.0	2.1	10.7	3.2	3.2	2.0	10.2	5.9	3.2	4.5	4.5
GABA	7.4	2.6	2.0	6.0	3.5	5.4	0.9	10.4	11.7	5.3	4.2	8.2
TMAO	11.7	11.3	2.0	10.2	3.9	8.6	2.6	9.5	11.0	5.9	4.0	7.8

NOR	4.6	6.4	17.0	12.0	7.9	3.1	7.9	9.8	3.4	4.4	4.6	3.9
EPI	5.0	7.6	5.5	13.5	3.4	6.6	4.4	5.9	11.8	11.6	10.8	11.0
VAL	2.8	2.5	2.5	4.7	2.1	2.1	1.4	5.8	1.9	1.5	2.1	1.8
PA	5.9	3.9	1.0	6.6	2.4	6.3	0.1	8.0	7.8	8.5	6.0	7.6
NA	1.0	1.1	1.7	4.2	1.3	1.3	1.6	6.2	7.0	5.9	6.4	6.6
MET-D_4	2.9	2.2	2.1	4.9	1.1	1.6	1.2	4.4	3.3	3.0	2.8	2.9
MET	7.1	11.1	5.1	8.9	3.8	3.7	3.0	13.5	3.5	3.9	8.2	6.6
QA	2.7	4.8	4.9	5.4	2.7	3.5	1.9	5.2	1.7	2.2	1.5	1.8
L-DOPA	5.5	3.9	3.6	5.2	4.6	4.4	2.8	5.4	2.7	4.0	3.0	3.2
2AM	3.6	4.6	3.0	7.2	2.8	3.5	1.0	11.5	8.2	8.2	7.4	10.1
DA-D_4	3.2	3.3	5.8	7.2	4.3	5.5	3.4	6.5	3.5	2.0	3.6	3.3
DA	1.7	2.8	3.1	6.6	6.4	4.7	2.9	6.7	2.9	2.4	2.3	2.7
3OH-KYN	3.5	2.2	2.2	4.2	2.8	2.6	4.2	4.7	1.3	2.0	1.6	1.7
ILE-D_{10}	1.7	1.5	3.0	5.0	1.7	2.2	1.3	5.7	4.0	3.1	3.1	3.6
ILE	5.3	8.0	13.1	11.1	8.3	10.8	6.5	12.3	5.1	7.5	3.8	5.7
TYR-D_4	1.9	1.8	2.7	4.1	2.5	3.3	0.6	5.9	1.5	1.4	1.9	1.8
TYR	2.8	2.3	2.3	6.2	3.7	2.8	0.1	6.3	2.2	1.8	7.5	4.4
TYRA	2.6	3.3	1.4	4.1	1.8	1.3	0.9	5.8	9.9	15.8	17.4	14.8
LEU-D_{10}	1.8	1.7	1.0	3.5	4.2	1.3	1.4	6.1	2.9	1.0	1.6	2.3
LEU	3.0	4.6	2.9	4.9	3.9	2.6	2.4	7.3	0.9	1.0	0.9	0.9
5-HT-D_4	5.9	9.0	3.4	8.7	8.8	10.1	4.7	9.3	6.0	5.6	6.3	6.0
5-HT	7.3	10.6	3.2	11.7	3.2	5.5	2.7	8.0	5.9	5.8	5.7	5.6
3ME-TYRA	2.8	4.8	3.5	6.3	2.4	2.4	4.5	3.5	2.4	3.4	3.4	3.0
5OH-TRP	5.7	11.9	2.2	10.8	2.8	8.3	1.3	6.9	9.4	14.1	15.6	14.3
ME-5HT	8.3	9.4	3.4	12.8	7.3	4.4	4.7	11.7	11.4	14.7	12.6	12.8
KYN-D_4	3.8	9.0	2.3	6.0	3.2	4.1	3.8	11.1	5.1	2.2	2.4	5.2
KYN	5.1	7.5	2.4	9.9	2.3	3.7	0.6	11.0	3.1	5.9	5.3	4.9
PHE	6.5	7.0	2.1	11.7	3.4	6.0	0.5	12.2	1.9	1.4	0.8	1.6
4OH-PAG	7.7	10.0	4.3	9.4	10.5	4.9	8.1	12.7	6.3	4.5	4.6	5.3
3OH-AA	3.0	4.0	2.8	5.7	3.4	3.0	0.4	8.1	14.4	13.5	12.5	13.6
TRP-D_5	1.5	4.2	3.1	11.7	2.9	2.7	2.0	7.7	2.5	5.3	5.6	4.5
XA-D_4	5.9	8.6	2.6	12.8	3.0	6.8	0.4	12.3	3.7	3.4	7.0	4.8
TRP	1.0	1.9	1.1	6.7	1.7	2.1	0.8	5.1	1.4	2.2	1.8	2.0

XA	3.0	5.2	5.3	5.1	1.9	2.7	0.7	3.0	2.6	3.9	2.0	3.0
DOPAC-D_5	7.2	8.5	6.6	9.2	4.5	4.3	6.1	8.7	2.0	2.1	2.1	2.0
DOPAC	8.1	6.3	12.0	8.4	8.9	9.2	8.6	8.9	4.2	3.3	3.4	3.9
4OH-PLA	6.4	9.6	6.2	11.4	5.8	3.5	6.8	6.6	3.8	4.5	3.6	4.2
HVAS	7.9	9.4	13.1	12.0	4.5	7.7	6.2	9.1	3.0	2.5	3.0	2.8
NAC-TYR	4.6	9.6	7.5	8.4	5.0	5.6	2.7	6.9	3.5	2.8	2.8	3.7
TROL	4.7	5.8	3.9	10.1	2.4	2.3	4.6	10.1	2.7	4.0	4.5	6.0
KA-D_5	1.7	4.7	1.8	12.4	1.5	4.0	2.8	6.6	0.9	1.5	1.5	1.7
KA	4.0	7.9	4.0	12.3	4.3	2.4	1.4	10.2	1.4	2.1	1.6	2.1
5ME-TRP	2.7	2.2	3.4	9.9	3.1	3.1	1.4	4.8	6.7	4.1	4.1	7.4
TRYT	8.4	9.9	3.7	9.6	4.0	5.1	1.8	10.6	1.1	2.2	1.0	1.6
4OH-PPG	2.6	2.6	2.4	3.3	3.5	2.0	2.4	3.7	3.2	2.1	2.0	3.0
5OH-IET	1.4	2.5	3.4	10.2	1.3	2.8	5.7	7.0	1.8	2.6	2.6	2.8
5ME-TRYT	1.7	4.4	2.6	6.4	1.3	2.6	1.8	2.9	2.7	2.6	2.6	2.6
6-SMEL	6.8	11.8	9.0	9.4	6.4	5.6	0.7	8.7	5.5	2.4	2.7	3.7
5OH-IAA-D_5	6.3	4.1	11.1	7.8	7.8	6.0	6.5	9.8	1.8	1.3	1.3	1.5
5OH-IAA	11.8	7.0	5.7	12.9	8.4	7.9	6.3	11.2	9.4	10.8	12.9	10.8
PLI	5.2	9.1	10.1	9.2	11.0	7.1	11.7	9.8	4.9	2.6	4.2	4.3
NAC-5HT	2.1	1.8	4.1	7.9	2.2	1.3	1.2	4.0	4.2	3.0	3.0	3.5
IBG	6.7	8.6	11.0	10.7	8.3	7.2	11.5	10.4	2.7	3.0	3.0	3.3
IS-D_4	2.9	4.2	3.9	4.9	1.8	4.2	0.4	4.2	1.3	1.3	2.6	4.2
IS	2.8	7.9	5.7	6.5	2.2	4.4	1.0	6.4	1.7	1.8	1.8	2.1
PAGLU	1.2	2.0	4.0	4.9	1.0	0.8	0.6	1.5	1.7	1.4	1.4	1.5
HIP-D_5	1.9	1.7	4.3	4.5	0.7	2.1	0.9	2.0	2.4	1.8	1.6	2.1
HIP	4.2	6.8	3.1	8.0	2.3	1.8	1.6	8.1	4.1	2.4	1.3	2.9
TRP ME	2.9	6.4	3.3	12.0	2.3	2.8	1.8	4.9	3.8	1.5	2.5	2.9
HVA	7.4	9.1	8.4	8.6	7.9	13.0	3.9	12.7	6.6	7.2	7.1	6.5
PAGLY	3.3	5.7	2.5	6.2	2.3	2.1	2.2	5.8	1.4	0.7	1.0	1.1
IASP	3.4	3.7	2.8	8.9	3.9	2.5	2.4	5.0	7.8	3.3	2.9	5.1
4OH-PPA	13.0	8.2	10.7	11.7	7.7	8.9	4.3	11.1	2.1	1.4	5.4	3.7
PCG	6.9	10.8	11.8	10.5	2.8	2.4	6.1	7.7	1.3	4.9	13.6	7.5
IACT	10.7	9.3	2.2	8.6	5.8	5.9	2.6	8.4	2.4	2.2	1.8	2.2
TRP EE	1.6	2.4	1.6	5.5	1.1	2.2	2.2	2.3	3.1	2.4	2.7	3.6

HCA	8.5	3.8	6.5	6.9	6.1	7.2	4.5	7.5	2.5	3.8	3.8	3.6
PCS	5.3	8.4	8.5	8.8	2.4	3.7	5.6	6.5	4.3	1.7	3.8	4.0
IGLUT	2.8	3.0	3.6	3.5	2.6	1.7	0.8	4.8	11.2	13.2	11.3	12.9
AA	3.6	7.3	3.8	10.0	5.9	6.1	5.5	12.9	3.5	1.9	2.7	3.2
NAC-PHE	4.5	6.5	5.4	6.6	1.9	2.3	2.5	6.9	2.4	4.0	3.8	3.6
PLA	5.2	7.1	7.9	6.9	2.6	2.2	1.0	6.1	3.3	3.2	3.0	3.7
NAC-TRP	8.7	10.8	8.2	11.7	6.6	6.5	2.3	9.2	6.8	4.0	4.1	5.4
ILA	1.1	2.0	2.0	4.0	2.3	0.9	0.3	3.2	10.7	10.3	10.4	10.1
NAC-TYR EE	3.7	2.9	2.6	3.3	1.6	3.0	0.9	4.9	2.5	1.6	1.6	1.9
PPG	1.4	2.8	2.8	6.8	0.8	2.2	2.5	7.4	1.7	2.1	1.3	1.8
IAG	2.5	8.1	3.2	10.5	2.7	3.1	1.7	10.0	12.5	10.6	5.9	10.6
ICA	11.6	8.9	1.0	11.4	4.7	3.1	3.1	13.6	6.6	6.6	6.7	6.9
CYG	1.5	1.5	1.5	1.9	1.3	1.7	2.6	3.5	5.2	6.8	6.0	5.8
ICARB	9.6	7.0	8.1	10.8	7.5	7.3	4.6	11.4	5.5	7.0	8.2	9.7
MEL	1.2	1.6	1.7	3.5	1.5	2.7	1.4	5.0	3.4	2.6	3.4	3.1
5ME-IET	1.6	1.2	1.4	2.2	1.9	1.5	0.9	1.6	4.6	4.4	1.8	3.9
5ME-IAA	2.0	3.1	1.0	6.7	1.1	1.4	3.4	6.1	0.7	1.8	1.8	3.0
BA	12.7	8.8	5.1	12.3	9.8	9.0	6.7	9.1	3.8	3.1	3.1	3.3
IAA-D_5	2.3	0.9	3.5	3.4	1.1	1.6	2.2	4.9	1.6	1.1	2.8	2.4
IAA	1.2	0.8	3.2	3.7	1.4	1.7	1.9	5.1	1.7	1.0	1.0	1.4
IET	1.5	1.8	2.7	4.7	0.8	1.3	0.6	3.0	4.9	2.6	4.2	4.3
CNBA	4.0	3.6	1.3	4.9	2.9	2.6	2.3	4.3	10.7	10.7	9.9	10.0
IACR	1.0	1.4	1.7	5.7	1.7	1.7	0.3	8.5	3.5	2.3	2.1	3.5
IPA	3.4	8.4	2.5	5.5	3.8	2.6	2.3	5.6	3.1	6.1	2.0	4.1
CA-D_5	3.5	10.6	2.9	8.5	4.0	5.0	6.1	9.9	3.0	2.5	3.0	2.8
CA	1.5	4.4	3.6	5.1	2.9	1.9	2.7	4.8	3.5	4.5	3.8	6.4
NAC-TRP EE	3.8	4.9	2.4	7.9	2.3	2.1	0.8	4.0	2.6	2.1	2.7	3.1
IBA	1.3	1.2	2.1	4.1	1.4	0.9	1.3	2.5	1.0	1.1	0.9	1.8
IACN	2.7	2.9	2.6	4.6	2.0	1.8	2.3	3.9	8.5	6.1	5.9	8.2
IAA ME	5.5	4.0	1.6	11.9	1.6	0.7	2.1	6.0	1.9	1.3	2.7	3.8
IND	2.7	4.0	3.1	4.8	2.7	2.5	1.8	4.5	12.1	9.3	4.2	9.7
IAA EE	2.3	4.0	2.1	3.9	1.5	3.3	1.5	3.4	1.9	2.3	2.3	2.1
TRPT	1.8	1.6	2.3	4.0	1.8	1.0	0.6	2.5	11.5	10.4	9.8	11.4

SKA	0.9	2.1	1.1	2.2	0.8	0.8	0.8	3.1	4.1	5.2	4.7	7.0

Supporting Table 8. Metabolite stability in aqueous solution (water 1 mM ammonium formate 0.5% formic acid) after 24, 48 and 72 hours in autosampler (5°C), expressed as calculated concentration (%) respect to time 0.

Name	SAMPLING TIME			
	24 H	48 H	72 H	CV%
Histamine	109.1	107.0	103.7	5.3
Histidine	102.4	99.6	98.7	5.3
TMA	116.0	102.7	109.7	6.9
GABA	90.9	103.0	95.3	7.0
TMAO	107.0	96.6	105.4	10.1
Norepinephrine	104.0	105.9	101.0	8.6
Epinephrine	106.6	107.5	109.9	7.1
L-valine	105.9	112.1	114.0	5.9
Picolinic acid	91.7	112.4	98.9	9.2
Nicotinic acid	101.7	109.9	107.6	4.2
L-methionine-D_4	105.0	99.8	111.2	5.2
L-methionine	99.0	108.9	113.8	9.1
Quinolinic acid	104.8	109.2	109.3	4.3
L-Dopa	101.8	100.4	106.9	4.9
2-aminophenol	108.3	106.4	117.9	7.3
Dopamine- D_4	109.8	105.9	108.1	6.1
Dopamine	109.9	108.6	108.2	5.6
3-hydroxykynurenine	104.0	111.1	110.6	4.8
L-isoleucine-D_{10}	110.0	114.4	116.3	5.5
L-isoleucine	96.7	102.9	98.1	7.0
L-tyrosine-D_4	105.3	109.1	110.7	4.1
L-tyrosine	104.8	99.9	109.0	4.2
Tyramine	105.5	109.4	106.5	3.5
L-leucine-D_{10}	98.4	103.7	103.7	3.3
L-leucine	104.5	110.8	109.5	4.3
Serotonin-D_4	103.5	103.9	95.9	6.6
Serotonin	110.2	97.6	114.6	10.3
3-methoxy-*p*-tyramine	107.1	93.9	95.1	6.5
5-hydroxy-L-tryptophan	97.0	96.9	96.0	7.7
N-methylserotonin	96.9	103.4	106.3	10.2

L-kynurenine-D_4	108.3	96.5	116.8	9.4
L-kynurenine	92.8	90.6	93.2	7.2
L-phenylalanine	104.5	105.3	114.1	6.6
4-hydroxyphenylacetylglycine	110.2	100.5	110.9	12.1
3-hydroxyanthranilic acid	101.7	109.4	107.3	4.6
L-tryptophan-D_5	106.0	100.5	104.5	5.8
Xanthurenic acid-D_4	105.2	107.8	106.3	7.2
L-tryptophan	105.5	101.0	111.5	4.7
Xanthurenic acid	102.5	103.5	99.9	4.6
3,4-dihydroxyphenylacetic acid-D_5	104.9	116.3	106.0	8.3
3,4-dihydroxyphenylacetic acid	109.6	109.6	100.8	9.4
3-(4-hydroxyphenyl)-lactic acid	93.5	102.5	102.1	7.6
Homovanillic acid sulfate	92.8	103.4	97.5	6.3
N-acetyl-L-tyrosine	95.0	111.3	102.5	7.8
L-tryptophanol	100.3	82.8	87.3	10.9
Kynurenic acid-D_5	91.6	86.1	89.9	7.2
Kynurenic acid	107.2	109.4	111.4	5.0
5-methoxy-L-tryptophan	101.3	91.3	100.4	5.0
Tryptamine	105.7	98.0	116.3	4.4
4-hydroxyphenylpropionylglycine	91.0	97.4	84.5	7.0
5-hydroxytryptophol	87.0	76.6	79.5	10.3
5-methoxytryptamine	103.4	95.7	94.4	5.2
6-sulfathoxymelatonin	93.1	95.2	95.9	7.0
5-hydroxyindole-3-acetid acid-D_5	97.5	95.4	104.0	13.4
5-hydroxyindole-3-acetid acid	100.1	103.8	107.8	7.2
Indoxyl-β-glucoside	86.2	106.6	95.3	11.8
N-acetyl-5-hydroxytryptamine	101.8	105.7	95.8	4.3
Indoxyl-β-glucuronide	100.9	103.9	103.4	7.4
Indoxyl sulfate-D_4	96.7	101.1	97.8	3.9
Indoxyl sulfate	94.5	103.4	96.4	4.3
Phenylacetyl-L-glutamine	101.6	96.4	100.6	2.8
Hippuric acid-D_5	100.1	98.0	98.5	1.8
Hippuric acid	101.8	107.8	102.8	4.3

L-tryptophan, methyl ester	94.2	89.2	90.8	4.8
Homovanillic acid	107.7	96.5	95.2	11.9
Phenylacetylglycine	100.8	107.2	100.8	3.2
Indole-3-acetyl aspartic acid	96.7	88.6	99.3	5.6
3-(4-hydroxyphenyl)-propionic acid	92.7	93.5	95.3	5.9
p-cresol glucuronide	92.0	91.5	92.5	5.6
Indole-3-acetamide	101.9	98.7	96.4	11.3
L-tryptophan, ethyl ester	99.7	98.9	98.5	1.4
4-hydroxycinnamic acid	94.4	97.7	93.3	6.2
p-cresol sulfate	96.7	99.3	97.9	5.4
Indole-3-acetylglutamic acid	96.9	102.1	96.2	3.2
Anthranilic acid	102.1	103.2	108.7	11.9
N-acetyl-L-phenylalanine	91.5	100.4	94.3	5.5
Phenyllactic acid	94.6	97.6	92.7	4.3
N-acetyl-L-tryptophan	105.8	107.1	97.7	7.7
Indole-3-lactic acid	99.4	98.8	99.8	1.7
N-acetyl-L-tyrosine, ethyl ester	95.3	101.9	90.8	5.4
Phenylpropionylglycine	90.6	107.2	86.3	9.4
Indole-3-acryloylglycine	96.8	95.8	93.6	3.8
Indole-3-carboxylic acid	96.3	102.1	104.0	9.5
Cinnamoylglycine	98.0	101.4	97.1	2.1
Indole-3-carboxaldehyde	107.1	102.0	97.7	12.0
Melatonin	94.1	104.0	82.6	9.2
5-methoxytryptophol	95.7	96.2	91.2	3.4
5-methoxyindole-3-acetic acid	96.3	109.7	93.1	6.9
Benzoic acid	103.5	99.0	93.1	11.1
Indole-3-acetic acid-D_5	97.4	99.2	98.7	2.1
Indole-3-acetic acid	95.6	98.1	97.1	2.1
Indole-3-ethanol	101.5	100.4	101.1	1.9
Cinnabarinic acid	94.7	94.8	90.0	11.1
Indole-3-acrylic acid	90.4	87.5	79.9	8.1
Indole-3-propionic acid	93.5	95.4	81.4	7.8
trans-cinnamic acid-D_5	92.0	95.0	91.7	4.6

trans-cinnamic acid	90.5	92.2	97.0	4.5
N-acetyl-L-tryptophan, ethyl ester	102.0	97.5	89.8	5.4
Indole-3-butyric acid	98.0	98.3	101.3	1.8
Indole-3-acetonitrile	98.2	97.1	101.8	2.7
Indole-3-acetic acid, methyl ester	97.2	98.9	89.3	5.0
Indole	97.6	97.7	88.6	5.4
Indole-3-acetic acid, ethyl ester	95.8	92.7	89.2	4.6
Tryptanthrin	87.0	79.0	74.9	11.0
3-methylindole	100.9	102.4	101.4	2.0

Supporting Table 9. Percentage coefficient of variation values for QC samples acquired to assess analytical performance during method validation.

Name	PLASMA	URINE
	CV%	CV%
MET-D_4	12.5	6.6
DA-D_4	5.0	3.5
ILE-D_{10}	6.5	3.9
TYR-D_4	4.8	4.1
LEU-D_{10}	6.1	2.6
5-HT-D_4	6.2	11.9
KYN-D_4	13.7	13.6
TRP-D_5	2.8	8.5
XA-D_4	14.4	9.9
DOPAC-D_5	5.0	7.6
KA-D_5	3.2	8.9
5OH-IAA-D_5	12.8	9.7
IS-D_4	9.0	5.0
HIP-D_5	5.3	3.2
IAA-D_5	2.4	3.3
CA-D_5	3.1	6.5

Supporting Table 10. Percentage coefficient of variation values for plasma (n = 16) and urine (n = 17) pooled QC samples from MUCOL study. Good system stability is indicated by % CV < 15%.

Name	PLASMA	URINE
	CV%	CV%
MET-D_4	4.0	5.3
DA-D_4	2.6	9.0
ILE-D_{10}	2.3	4.8
TYR-D_4	2.6	5.4
LEU-D_{10}	2.6	3.4
5-HT-D_4	7.9	9.9
KYN-D_4	8.7	9.9
TRP-D_5	11.8	12.1
XA-D_4	8.4	12.2
DOPAC-D_5	12.1	25.5
KA-D_5	5.8	13.1
5OH-IAA-D_5	8.3	10.9
IS-D_4	9.0	8.0
HIP-D_5	6.2	3.3
IAA-D_5	2.3	4.8
CA-D_5	4.0	10.4

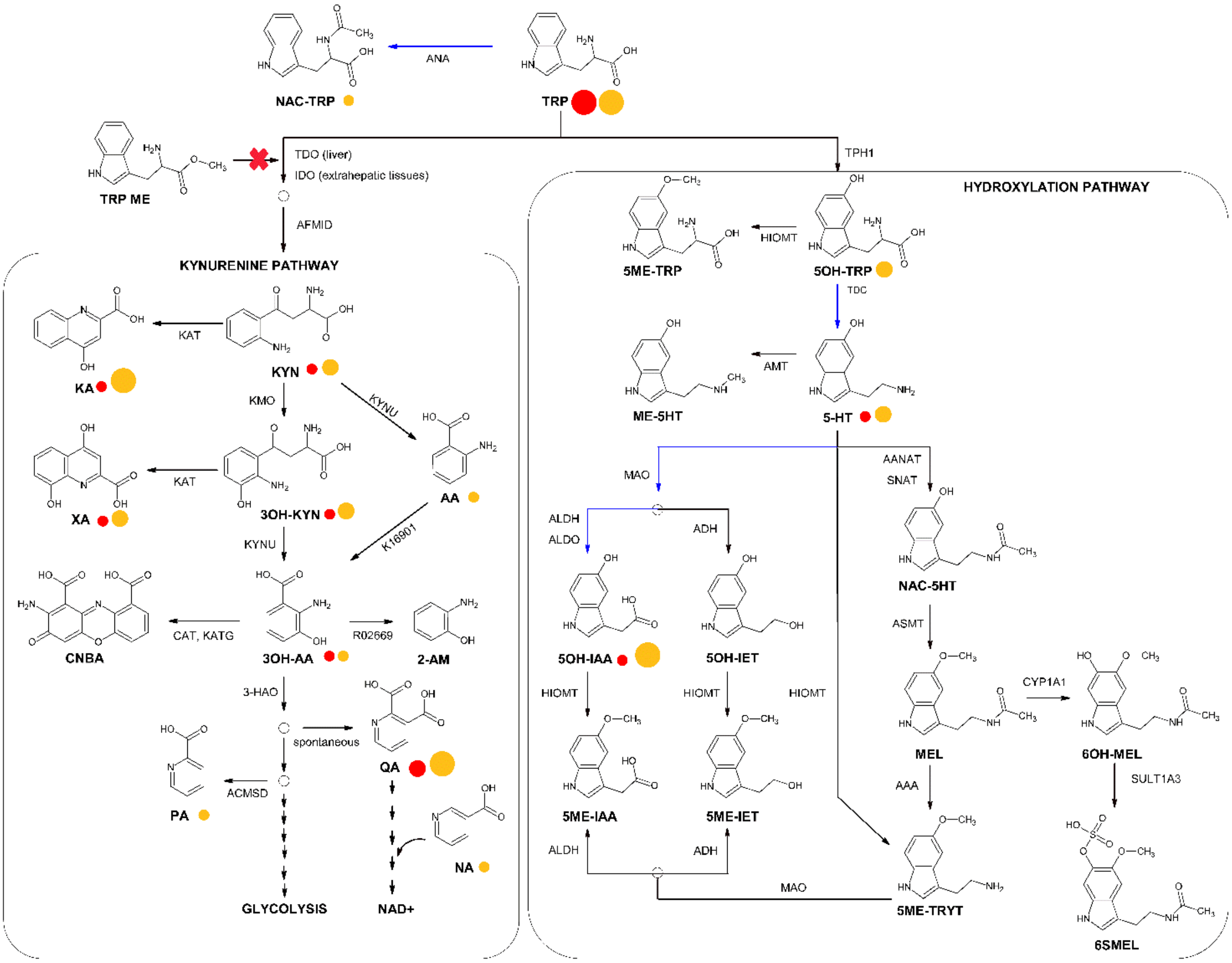

LEGEND: ◌ : metabolite with median concentration < 1 mM; ◌ :median concentration 1 < x < 10 mM ◌ : median concentration 1 < x < 10 mM

● : metabolite detected in plasma; ● : metabolite detected in urine

→ : host metabolism; → : microbial metabolism; → :host-microbial co-metabolism; - - → :unknown enzyme

ANA: arylamine N-acetyltransferase (EC2.3.1.5)
TDO: tryptophan 2,3-dioxygenase (EC 1.13.11.11)
IDO: indolamine 2,3-dioxygenase (EC 1.13.11.52)
AFMID: arylformidase (EC 3.5.1.9)
KAT: kynurenine aminotransferases (EC 2.6.1.7)
KMO: kynurenine 3-monooxygenase (EC 1.14.13.9)
KYNU: kynureninase (EC 3.7.1.3)
K16901: anthranilate 3-monooxygenase (EC 1.14.14.8)
CAT: catalase (EC 1.11.1.6)
KATG: catalase-peroxidase (EC 1.11.1.21)
3-HAO: 3-hydroxyanthranilic acid 3,4-dioxygenase (EC 1.13.16.1)
R02669: decarboxylase (EC 4.1.1.-)
ACMSD: 2-a,mino-3-carboxymuconic-6-semialdehyde decarboxylase (EC 4.1.1.45)

TPH1: tryptophan-5-monooxygenase (EC 1.14.16.4)
ASMT/HIOMT: acetylserotonin (hydroxyindole) O-methyltransferase (EC 2.1.1.4)
TDC: tryptophan decarboxylase (EC 4.1.1.28)
AMT: amine N-methyltransferase (EC 2.1.1.49)
MAO: monoamine oxidase (EC 1.4.3.4)
ALDH: aldehyde dehydrogenase (EC 1.2.1.3)
ALDO: aldehyde oxidase (EC 1.2.3.1)
ADH: alcohol dehydrogenase (EC 1.1.1.1)
AANAT: arylalkylamine N-acetyltransferase (EC 2.3.1.87)
SNAT: arylamine N-acetyltransferase (EC 2.3.1.5)
AAA : aryl-acylamidase (melatonin deacetylase) (EC 3.5.1.13)
CYP1A1: cytochrome P450 family subfamily A polypeptide 1 (EC 1.14.14.1)
SULT1A3: PAPS-dependent amine sulfotrnasferase (EC 2.8.2.3)

Supporting Figure S1. Tryptophan catabolic pathway through kynurenines (left) and hydroxylation pathway (right). NAC-TRP: *N*-acetyl-tryptophan; TRP: L-tryptophan; TRP ME: tryptophan methyl ester; KA: kynurenic acid; KYN: L-kynurenine; XA: xanthurenic

acid; 3OH-KYN: 3-hydroxykynurenine; AA: anthranilic acid; CNBA: cinnabarinic acid; 3OH-AA: 3-hydroxyanthranilic acid; 2-AM: 2-aminophenol; PA: picolinic acid; QA: quinolinic acid; NA. nicotinic acid; 5ME-TRP: 5-methoxy-L-tryptophan; 5OH-TRP: 5-hydroxy-L-tryptophan; 5-HT: serotonin; ME-5HT: *N*-methylserotonin; 5OH-IAA: 5-hydroxyindole-3-acetic acid; 5ME-IAA: 5-methoxyindole-3-acetic acid; 5OH-IET: 5-hydroxytryptophol; 5ME-IET: 5-methoxytryptophol; NAC-5HT: *N*-acetyl-5hydroxytryptamine; MEL: melatonin; 6OH-MEL: 6-hydroxymelatonin; 5ME-TRYT: 5-methoxytryptamine; 6-SMEL: 6-sulfatoxymelatonin. Green arrows represent gut microbial metabolism; black arrows represent host metabolism and blue arrows represent host-microbiota metabolism.

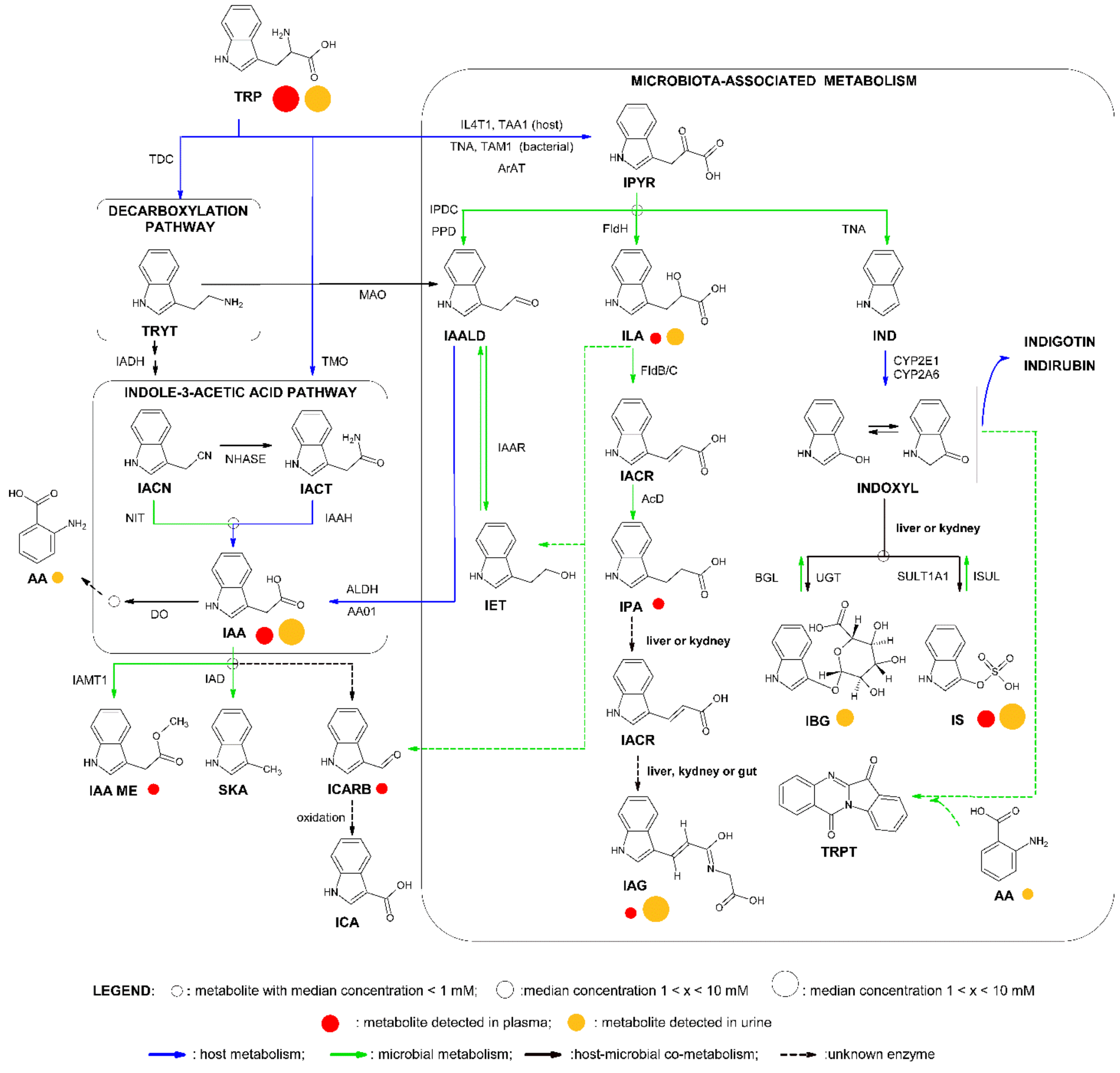

TDC: tryptophan decarboxylase (EC 4.1.1.28)
MAO: monoamine oxidase (EC 1.4.3.4)
IADH: indoleacetaldoxime dehydratase (EC 4.99.1.6)
TMO: tryptophan monooxygenase (EC 1.13.12.3)
NHASE: nitrile hydratase (EC 4.2.1.84)
NIT: nitrilase (EC 3.5.5.1)
DO: dioxygnese (EC 1.13.11.-)
IAAH: indoleacetamide hydroxylase (EC 3.5.1.4)
IAMT1: indole-3-acetate-O-methyltransferase (EC 2.1.1.278)
IAD: indoleacetate decarboxylase (EC 4.1.1.115)
IL4T1: L-amino acid oxidase (EC 1.4.3.2)
TAA1: L-tryptophan-pyruvate aminotransferase (EC 2.6.1.29)
ArAT: aromatic amino acid aminotransferase (EC 2.6.1.57)
TNA: tryptophanase (EC 4.1.99.1)

TAM1: tryptophan aminotransferase (EC 2.6.1.27)
IPDC: indole pyruvate decarboxylase (EC 4.1.1.74)
PPD: phenylpyruvate decarboxylase (EC 4.1.1.43)
ALDH: aldehyde dehydrogenase (EC 1.2.1.3)
AA01: indole-3-acetaldehyde oxidase (EC 1.2.3.7)
IAAR: Indoleacetaldehyde reductase (EC 1.1.1.190)
FldH: phenyllactate dehydrogenase (EC 1.1.1.110)
FldB/C: phenyllactate dehydratase subunits B/C (EC 4.2.1.175)
AcD: Acyl-CoA dehydrogenase (EC 1.3.8.1)
CYP2E1: cytochrome P450 2E1 (EC 1.14.13.n7)
CYP2A6: cytochrome P450 2A6 (EC 1.14.14.1)
BGL: B-glucosidase (EC 3.2.1.21)
UGT: UDP-glucuronyltransferase (EC 2.4.1.-)
SULT1A1: PAPS-dependent aryl sulfotrnasferase (EC 2.8.2.1)
ISUL: Indoxyl sulfatase (EC 3.1.6.-)

Supporting Figure S2. Microbiota associated tryptophan catabolism. TRP: L-tryptophan; TRYT: tryptamine; IACN: indole-3-acetonitrile; IACT: indole-3-acetamide; AA: anthranilic acids; IAA: indole-3-acetic acids; IAA ME: indole-3-acetic acid methyl ester; SKA: 3-methyl indole (skatole); ICARB: indole-3-carboxaldehyde; ICA: indole-3-carboxylic acid; IPYR: indole-3-pyruvic acid; IAALD: indole-3-acetaldehyde; IET: indole-3-ethanol; ILA: indole-3-lactic acid; IACR: indole-3-acrylic acid; IPA: indole-3-propionic acid; IAG: indole-3-acryloylglycine; IND: indole; IBG: indoxyl-β-glucuronide; PLI: indoxyl- β-glucoside (plant indican); IS: indoxyl sulfate; TRPT: tryptanthrin. Green arrows represent gut microbial metabolism; black arrows represent host metabolism, blue arrows represent host-microbiota metabolism while dashed arrows represent unknown reactions.

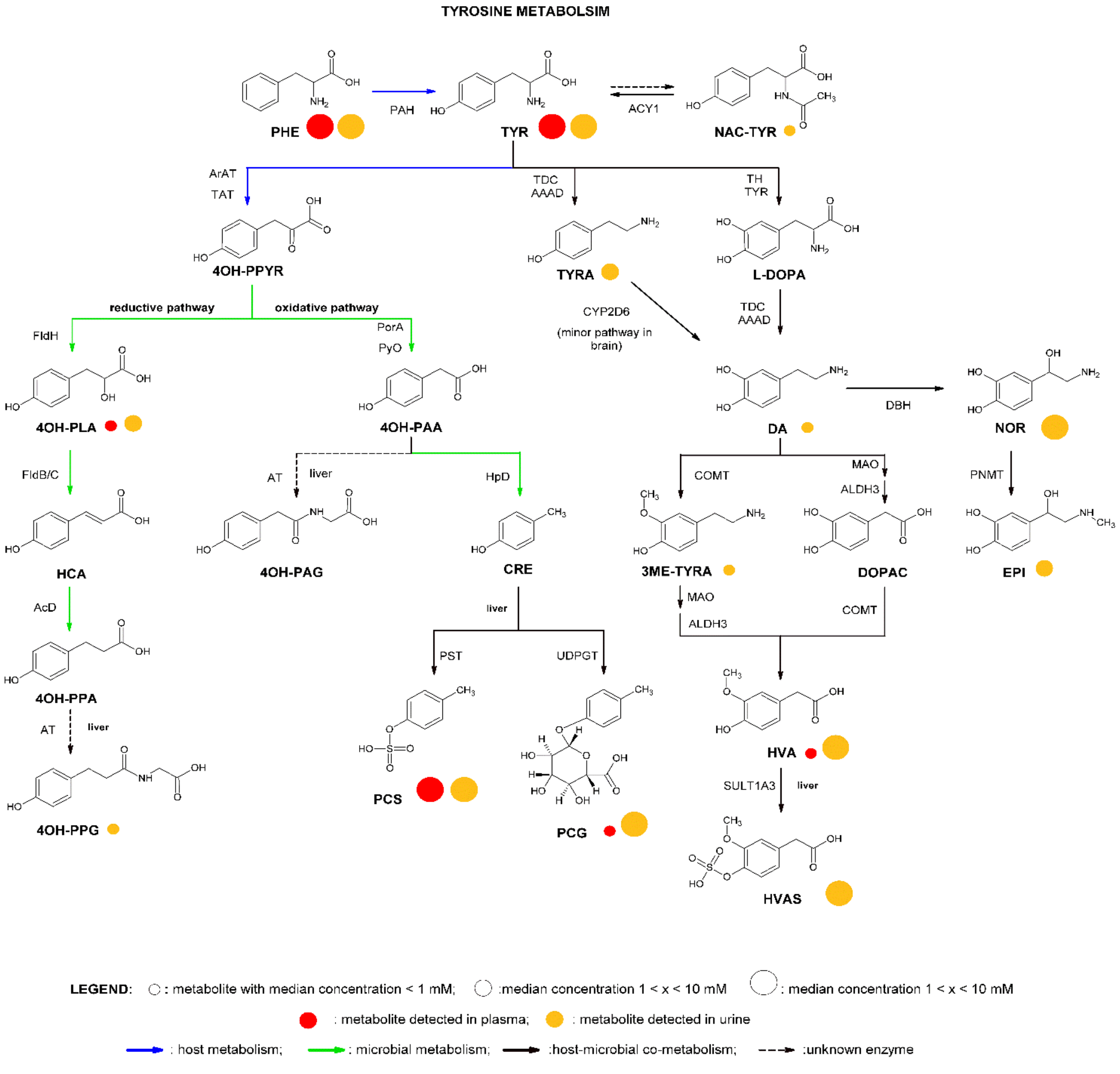

PAH : phenylalanine-4-hydroxylase (EC 1.14.16.1)
ACY1: aminoacylase 1 (EC 3.5.1.14)
ArAT: aromatic amino acid aminotransferase (EC 2.6.1.57)
TAT: tyrosine aminotransferase (EC 2.6.1.5)
FldH: phenyllactate dehydrogenase (EC 1.1.1.110)
FldB/C: phenyllactate dehydratase subunit B/C (EC 4.2.1.175)
AcD: acyl-CoA dehydrogenase (EC 1.3.8.1)
PorA: pyruvate:ferredoxin oxidoreductase A (EC 1.2.7.1)
PyO: 4-hydroxyphenylpyruvate oxidase (EC 1.2.3.13)
AT: acyltransferase (EC 2.3.1.-)
HpD: hydroxyphenylacetate decarboxylase (EC 4.1.1.83)
PST: aryl sulfotransferase(EC 2.8.2.1)
UDPGT: UDP-glucuronyltransferase (EC 2.4.1.17)

TDC: tyrosine decarboxylaase (EC 4.1.1.25)
AAAD: aromatic-L-aminoacid/L-tryptophan decarboxylase (EC 4.1.1.28)
CYP2D6: Cytochrome P450 2D6 (EC 1.14.14.1)
TH: tyrosine 3-monooxygenase (EC 1.14.16.2)
TYR: tyrosinase (EC 1.14.18.1)
DBH: dopamine beta-monooxygenase (EC 1.14.17.1)
PNMT: phenylethanolamine N-methyltransferase (EC 2.1.1.28)
COMT: catechol O-methyltransferase (EC 2.1.1.6)
MAO: monoamine oxidase (EC 1.4.3.4)
ALDH3: aldehyde dehydrogenase (NAD(P)+) (EC 1.2.1.5)
SULT1A3: sulfotransferase 1A3 (EC 2.8.2.1)

Supporting Figure S3. Tyrosine catabolic pathway. PHE: L-phenylalanine; TYR: L-tyrosine; NAC-TYR: *N*-acetyl-L-tyrosine; 4OH-PPYR: 4-hydroxyphenylpyruvic acid; 4OH-PLA: 4-hydroxyphenyllactic acid; HCA: 4-hydroxycinnamic acid; 4OH-PPA: 4-hydroxyphenylpropionic acids; 4OH-PAA: 4-hydroxyphenylacetic acid; 4OH-PPG: 4-hydroxyphenylpropionylglycine; 4OH-PAG: 4-hydroxyphenylacetylglycine; CRE: *p*-cresol; PCS: *p*-cresol sulfate; PCG: *p*-cresol glucuronide; TYRA: tyramine; L-DOPA: L-Dopa; DA: dopamine; NOR: norepinephrine; EPI: epinephrine; 3ME-TYRA: 3-methoxy-*p*-tramine; DOPAC: 3,4-dihydroxyphenylacetic acid; HVA: homovanillic acid; HVAS: homovanillic acid sulfate. Green arrows represent gut microbial metabolism; black arrows represent host metabolism and blue arrows represent host-microbiota metabolism.

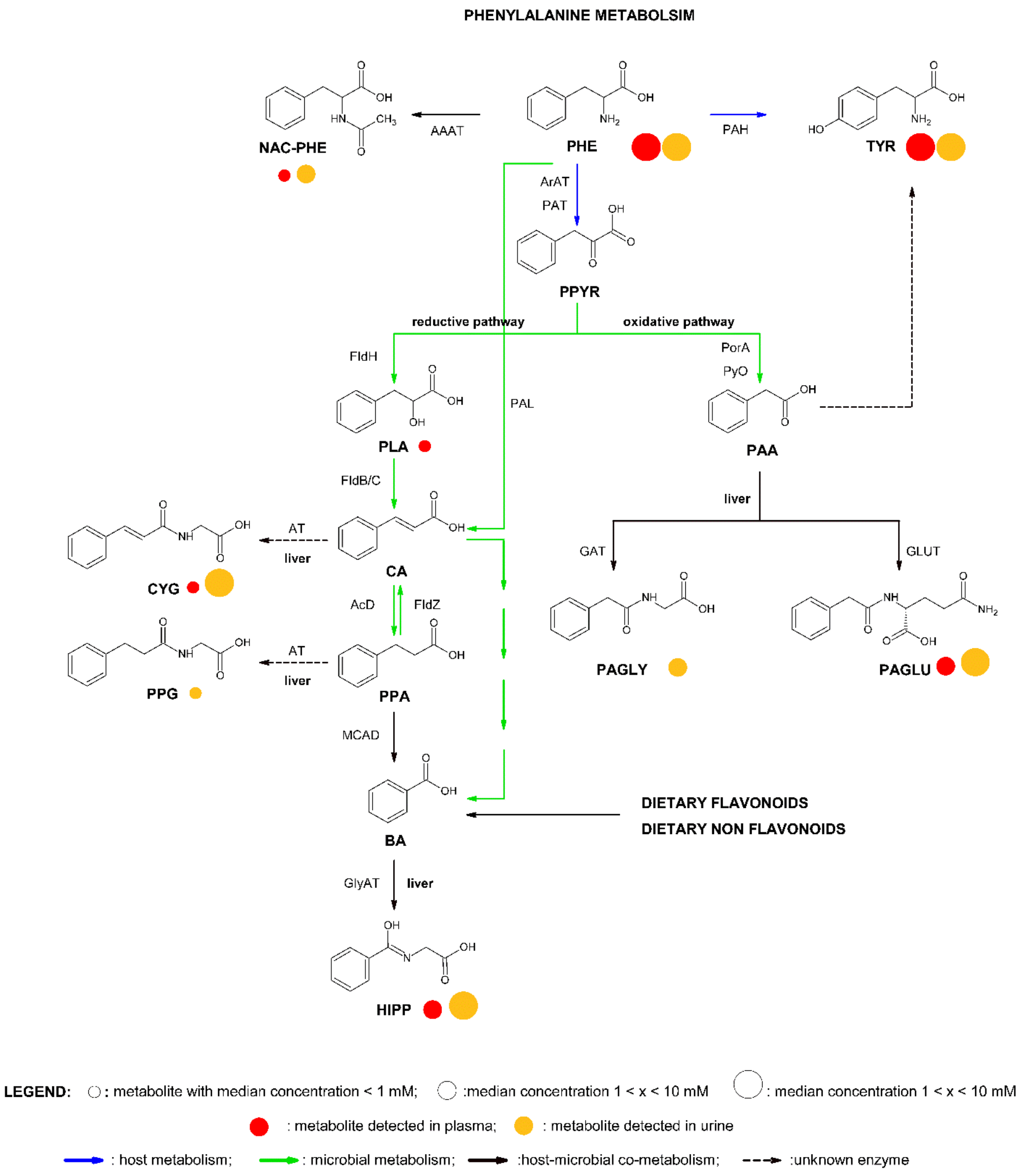

AAAT:L-phenylalanine/L-methionine-N-acetyltransferase (EC 2.3.1.53)
PAH : phenylalanine-4-hydroxylase (EC 1.14.16.1)
ArAT: aromatic amino acid aminotransferase (EC 2.6.1.57)
PAT: phenylalanine (histidine) transaminase (EC 2.6.1.58)
FldH: phenyllactate dehydrogenase (EC 1.1.1.110)
FldB/C: phenyllactate dehydratase (EC 4.2.1.175)
AcD: Acyl-CoA dehydrogenase (EC 1.3.8.1)
FldZ: cinnamate reductase (EC 1.3.1.-)
MCAD: medium chain acyl-CoA dehydrogenase (EC 1.3.8.7)
AT: acyltransferase (EC 2.3.1.-)
GlyAT: glycine N-benzoyltransferase (EC 2.3.1.71)
PorA: pyruvate:ferredoxin oxidoreductase A (EC 1.2.7.1)
PyO: 4-hydroxyphenylpyruvate oxidase (EC 1.2.3.13)
PAL: phenylalanine ammonia lyase (EC 4.3.1.24)
GAT: glycine N-phenylacetyltransferase (EC 2.3.1.192)
GLUT: glutamine N-phenylacetyltransferase (EC 2.3.1.14)

Supporting Figure S4: Phenylalanine catabolic pathway. NAC-PHE: *N*-acetyl-L- phenylalanine; PHE: L-phenylalanine; TYR: L-tyrosine; PPYR: phenylpyruvic acid; PLA: phenyllactic acid; CYG: cinnamoylglycine; CA: cinnamic acid; PPG: phenylpropionylglycine; PPA: phenylpropionic acids; BA: benzoic acid; HIP: hippuric acid; PAA: phenylacetic acid; PAGLY: phenylacetylglycine; PAGL: phenylacetylglutamine.

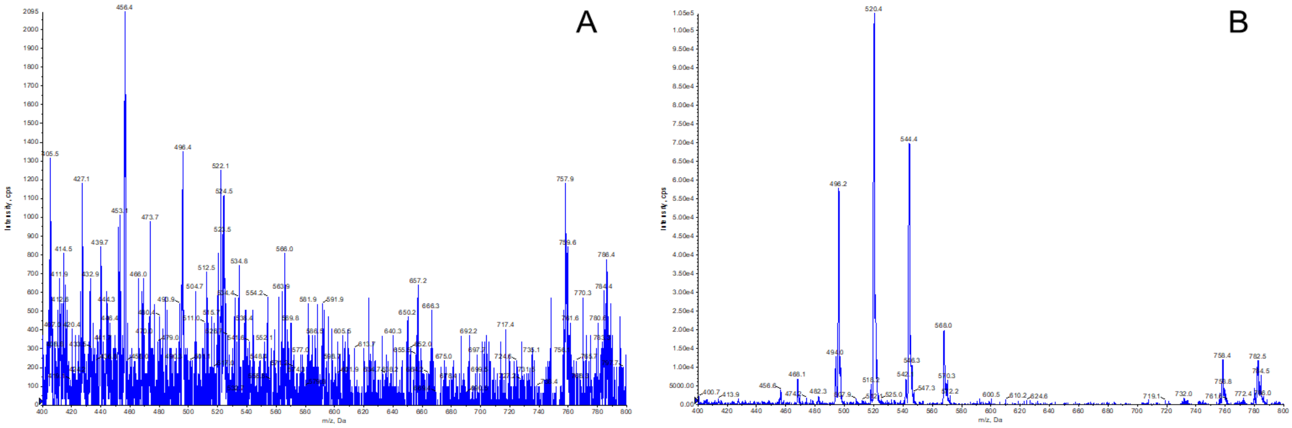

Supporting Figure S5. Precursor Ion Scanning (PIS) of 184.3 (protonated phosphocholine of phosphatidylcholine and sphingomyelin) in second extraction step using ACN 1% FA (panel A) or MeOH 1% ammonium formate (B). Lyso-PC and PC are retained on Ostro 96-well plate sorbent when ACN 1% FA is employed. Significant amount of lyso-PC 16:0 (*m/z* 496.2), lyso-PC 18:2 (*m/z* 520.4), lyso-PC 20:4 (*m/z* 544.4) and lyso-PC 22:6 (*m/z* 568.4), PC 34:2 (*m/z* 758.4), PC 36:4 (*m/z* 782.5) and PC 36:3 (*m/z* 784.5) are detectable when using MeOH 1% ammonium formate.

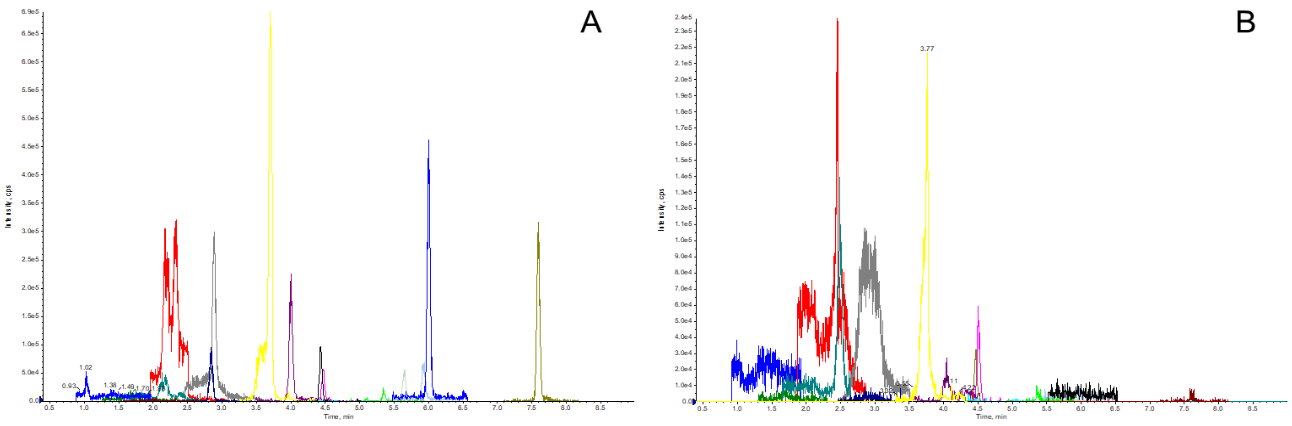

Supporting Figure S6. Extracted Ion Chromatogram (XIC) of VAL, MET, ILE, LEU, TYR, PHE, KYN, TRP, KA, HIP, IAA, ILA, CINNA and TRPT in A) well plate washed with MeOH after one-single reconstitution step and B) well plate washed with MeOH after performing two washings with reconstitution solvent. Only traces of TRP are detectable in well plates after performing a two-step washing.

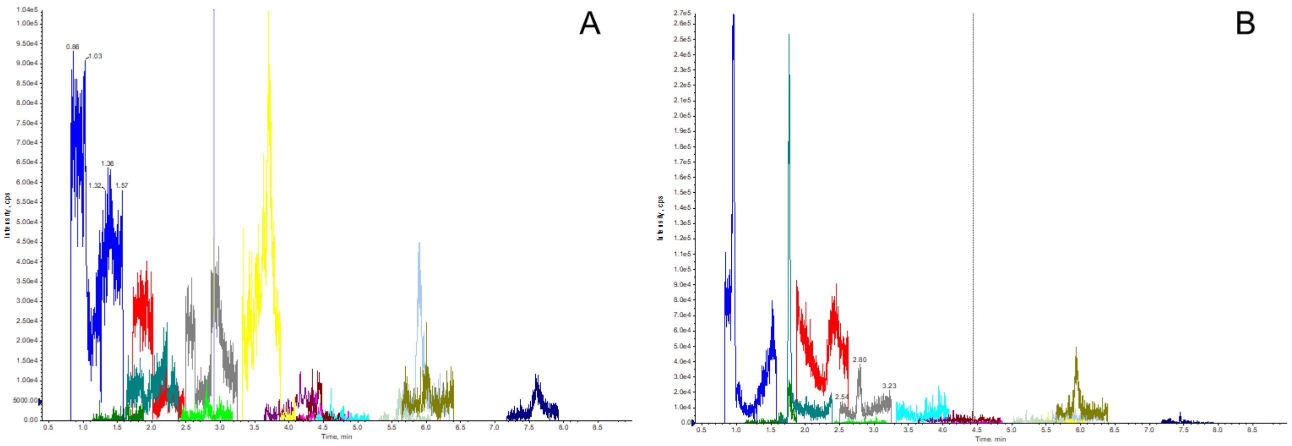

Supporting Figure S7. XIC of VAL, MET, ILE, LEU, TYR, PHE, KYN, TRP, KA, HIP, IAA, ILA, CINNA and TRPT of A) injection of MeOH after acquisition of the highest point of plasma and serum calibration curves and B) injection of MeOH after acquisition of a batch of solvent (*n*=7) and plasma samples (*n*=7) spiked at the highest metabolite concentration for recovery determination.
